# Rapid review of COVID-19 epidemic estimation studies for Iran

**DOI:** 10.1186/s12889-021-10183-3

**Published:** 2021-02-01

**Authors:** Farshad Pourmalek, Mohsen Rezaei Hemami, Leila Janani, Maziar Moradi-Lakeh

**Affiliations:** 1 University of British Columbia, Vancouver, Canada; 2 Aberdeen Centre for Health Data Sciences, University of Aberdeen, Aberdeen, UK; 3 Department of Biostatistics, School of Public Health, Iran University of Medical Sciences, Tehran, Iran; 4 Preventive Medicine and Public Health Research Center, Psychosocial Health Research Institute, Community and Family Medicine Department, School of Medicine, Iran University of Medical Sciences, Tehran, Iran

**Keywords:** COVID-19, Epidemic, Pandemic, Iran, Cases, Deaths, Model, Prediction, Estimation

## Abstract

**Background:**

To inform researchers about the methodology and results of epidemic estimation studies performed for COVID-19 epidemic in Iran, we aimed to perform a rapid review.

**Methods:**

We searched for and included published articles, preprint manuscripts and reports that estimated numbers of cumulative or daily deaths or cases of COVID-19 in Iran. We found 131 studies and included 29 of them.

**Results:**

The included studies provided outputs for a total of 84 study-model/scenario combinations. Sixteen studies used 3–4 compartmental disease models. At the end of month two of the epidemic (2020-04-19), the lowest (and highest) values of predictions were 1,777 (388,951) for cumulative deaths, 20,588 (2,310,161) for cumulative cases, and at the end of month four (2020-06-20), were 3,590 (1,819,392) for cumulative deaths, and 144,305 (4,266,964) for cumulative cases. Highest estimates of cumulative deaths (and cases) for latest date available in 2020 were 418,834 on 2020-12-19 (and 41,475,792 on 2020-12-31). Model estimates predict an ominous course of epidemic progress in Iran. Increase in percent population using masks from the current situation to 95% might prevent 26,790 additional deaths (95% confidence interval 19,925–35,208) by the end of year 2020.

**Conclusions:**

Meticulousness and degree of details reported for disease modeling and statistical methods used in the included studies varied widely. Greater heterogeneity was observed regarding the results of predicted outcomes. Consideration of minimum and preferred reporting items in epidemic estimation studies might better inform future revisions of the available models and new models to be developed. Not accounting for under-reporting drives the models’ results misleading.

**Supplementary Information:**

The online version contains supplementary material available at 10.1186/s12889-021-10183-3.

## Background

“On 31 December 2019, the World Health Organization (WHO) China Country Office was informed of cases of pneumonia unknown etiology (unknown cause) detected in Wuhan City, Hubei Province of China” [1]. The disease was officially designated as coronavirus disease 2019 or COVID-19 by WHO on 2020-02-11 [2]. Due to the rapid outbreak of the disease worldwide, WHO characterized the situation as a pandemic on 11 March [3]. The first two confirmed cases of COVID-19 in Iran were officially reported on 2020-02-19 in city of Qom, by the Ministry of Health and Medical Education (MOHME) (via [4, 5]). Since then, MOHME has officially reported number of cumulative and new confirmed cases, deaths, and recovered cases in a daily basis on press conferences. Those numbers are available by date on different web pages of the web site of the MOHME but are not compiled in one page. To our best knowledge, the most straightforward route to access cumulative and daily deaths and cases is the compilation of WHO situation reports or more comprehensive sources such as Johns Hopkins University dashboard for COVID-19 [4, 5]. Web site of Iranian MOHME was not accessible to us from Canada and United States most of the time, with an “Access Forbidden” massage from the “Security Department” of MOHME at several occasions.

Number of deaths and cases, in addition to other characteristics of epidemic are very important in decision-making and disease control. However, official reports suffer from undercounting in all countries. A relatively high percentage of patients with COVID-19 are asymptomatic or have a mild form of the disease which increases the chance of remaining undiagnosed. The emerging nature of COVID-19 has aggravated undercounting, as many countries are not prepared for conducting enough tests; as a result, many of suspected cases or deaths may not ever be confirmed by standard laboratory tests. Epidemiological studies of COVID-19 and model-based predictions and estimations are useful in assessing transmission rates, predicting epidemic trends and fatality rates with the inclusion of different intervention, environmental (seasonality), and virologic (mutations) scenarios, and thus can help policymakers for informed decision making in a timely way [6].

Despite the potential role of epidemic modeling and estimation studies in predicting outbreak size and trend, multiplicity of factors influencing viral disease transmission, relative uncertainty of data on model parameters, shifting disease dynamics in the setting of evolving epidemic, and suboptimality of model building methods and reporting, have been known to limit predictive models’ usefulness. A systematic review of individual-level prediction models for covid-19 concluded that many of the models suffer from poor reporting, high risk of bias, and optimistic reporting of performance [7].

Since the beginning of the outbreak in Iran, researchers inside and outside the country have used models to estimate or predict the size and trajectory of the epidemic of COVID-19 in Iran. All of them are introduced later in the methods and results section of this article. Some of these studies have been published in scientific journals, or available in their not yet peer-reviewed form or are presented as official or unofficial reports. We aimed to perform a rapid review mainly describing currently available COVID-19 estimates for Iran. We did not intend to scrutinize or criticize the studies or models at this point. Our objective was to review methods and results of COVID-19 epidemic estimation studies for Iran. The ultimate goal is to inform the audience, policy makers and researchers, for better decisions, as well as potential updates of their prediction or estimation studies or new studies being designed and conducted currently and in future.

## Methods

### Study design and outcomes of interest

This a rapid review, not a systematic review. The main outcomes of interest were the predicted values (and calendar dates) of (1) cumulative deaths, (2) cumulative cases, (3) daily deaths, and (4) daily cases of COVID-19 in Iran. Deaths are less dependent on testing than cases. Cumulative estimates do not show daily fluctuations. Daily estimates help demonstrate epidemic waves and peaks.

### Place and time scope of target studies

We included all studies which their target population was or included Iran, and we found them from 2020-03-19 to 2020-04-12. We updated our search from 2020-10-02 to 2020-10-05.

### Search strategy and selection criteria

There is not a customized protocol for the items that must be available in a report of epidemic estimation, prediction or epidemic model. Carrasco et al. proposed a ‘CCPV’ protocol (Characteristics, Construction, Parameterization and Validation aspects protocol) to standardize the reporting of Influenza pandemic models [8]. The items included in the “transparent reporting of a multivariable prediction model for individual prognosis or diagnosis” (TRIPOD) gives a general idea of the reporting items, however it is not specifically for the reason of epidemic prediction/ estimation models [9]. Wynants et al. review of prediction models for diagnosis and prognosis of covid-19 infection, even though focusing the individual-level modeling, can be considered in reporting epidemic estimation studies [7].

Based on our understanding of the TRIPOD statement, epidemic modelling literature, and the studies we reviewed, we think an epidemic estimation / prediction model is expected to report at least following items, that we call them ‘preferred reporting items’: (1) Epidemic start date and rationale, (2) Epidemic (disease) model type and description, (3) Statistical model type, description, and equation(s), (4) Model assumptions and their verification, (5) Model scenarios’ detailed description, (6) Validation process and findings, (7) List and sources of model parameters and input data, and (8) Model outputs preferably with uncertainty intervals for scenarios. Some of the reports that we found in our searching process were not the final versions; we included any study if met all the following ‘minimum reporting items’: (1) Provided estimates for at least one of the COVID-19 four outcomes of interests (cumulative deaths, cumulative cases, daily deaths or daily cases) in Iran in any period of time, (2) Provided a list of input data and their sources, (3) An explanation on methods of using input data and generation of model outputs was available.

Exclusion criteria were: (1) Absence of all four main outcomes of interest, or (2) Absence of the minimum reporting items, or (3) Elaboration on a previous modeling or estimation study without the aim (or content) of updating or improving the previous estimates.

By “report”, we mean studies results of which were not published as a journal article (or pre-print), but were released as short or long reports, available on the internet or shared with researchers. There are differences between epidemic modeling, prediction, and estimation; Modeling studies use explicit disease models and statistical models. Prediction studies do not use explicit disease models but predict (project) the number of cases and or deaths in future. Estimation studies provide estimates of cases for a recent point in time. The common feature of all these three study types is that they provide estimates of cases and or deaths in at least one point in calendar time. For pragmatic reasons, we call all of them as estimation studies.

Study revisions / updates: We actively searched and checked for revisions or updates of studies and their published formats (from pre-print, to journal pre-proof, to final published article). International studies update their estimations on a periodical basis: (1) DELPHI (Differential Equations Leads to Predictions of Hospitalizations and Infections) Epidemiological Case Predictions (DELPHI) [10], (2) Youyang Gu (YYG) [11], (3) Institute for Health Metrics and Evaluation (IHME) [12], (4) Imperial College (Imperial) [13], (5) Los Alamos National Laboratory (LANL) [14], and (6) Srivastava [15]. We stopped using new estimate updates on 2020-10-11, when we used the latest estimate by LANL (Los Alamos National Laboratories) [14]. As such, the latest estimation dates for international we finally included in our review are as follows (references are for studies’ data web sites): 2020-07-18 for DELPHI [16], 2020-10-05 for Gu (YYG) [17], 2020-10-09 for IHME [18], 2020-10-06 for Imperial College [19], 2020-10-11 for LANL [20], and 2020-09-12 for Srivastava [15]. We found a web site [21] and a published article [22] by Sabri and colleagues. We refer to them, as ‘Saberi (web site)’ and ‘Saberi (article)’ respectively. For the former, that included periodical updates, we used their 2020-03-30 version. Updates to their previous model [21] were later discontinued.

We searched PubMed and used Google Scholar and plain Google for articles (or reports) matching our study inclusion criteria. The used keywords were Iran, COVID, COVID-19, COVID 19, Corona, SARS-CoV-2, epidemic, outbreak, pandemic, case*, death*, fatal*, mortalit*, model*, estimat*, and predict*. The search syntax used in PubMed is shown in the Appendix. We performed the same search with keywords in Farsi in Google Scholar, Google, Scientific Information Database (of Iran) [23], and MAG-IRAN [24].

We also used studies or reports provided to us by our researcher colleagues. We report all the found, included, and excluded, studies using PRISMA 2009 flow diagram (Moher et al. 2009) [25] in Appendix Figure 1.

### Data abstraction methods

We developed a spreadsheet for abstracting the items of methods and results from included studies – the items not restricted to the minimum required ones. Each study was reviewed independently by at least two authors, and discrepancies were resolved with involvement of a third reviewer. Two reviewers (MML and LJ) finalized the abstracted items for methodology of the target studies.

For abstraction of the results of the studies, we selected a set of six fixed calendar dates, and found and recorded the estimated / predicted values of main outcomes (cumulative deaths, cumulative cases, daily deaths, or daily cases) for each of those dates. To start with, we fixed the presumed epidemic start time on the date on which the first two cases were officially reported dead, on 2020-02-19 (1398-11-30 Hijri solar), although later official reports indicated the actual start date of the epidemic to be earlier. Rationale for this was that most of the studies used the official reports to start with, and most of the studies’ predictions also started from that date (2020-02-19).

We decided not to include the estimations for the end of the first, second and third weeks of the epidemic in our set of fixed dates, since we were already in month two of the epidemic, for sake of brevity, and considering the less robust nature of the predictions as early as the first month when the numbers were much smaller. The set of six fixed calendar dates were designated as the end of each Hijri solar month after the epidemic presumed start date, since the start date coincided with the last day of month 11 of the Hijri solar calendar, and as such, targeting the end of each solar month would enhance cross-study comparisons and further use of administrative data. We did not report predictions beyond the month six, for the sake of brevity, and given the more uncertainty regarding such longer-term intervention scenarios and outcomes. However, we demonstrated all the time span of the available predictions in our graphs to provide a visual overview. 

Most of the reviewed studies had considered more than one scenario for the progression of the epidemic, based on different intervention options, and we treated them as study-scenarios. Some studies considered different statistical models for prediction of epidemic progress, and we treated them as study-models. Altogether, such arrangement provided multiple study-scenarios/models for which we abstracted the estimated / predicted values of the main outcomes. Occasional studies provided confidence limits for point estimates which were also recorded. Along with update of our search in October 2020, we included estimated values for latest dates available in 2020 (e.g. 2020-12-31) and in 2021 (e.g. 2021-01-31) where available.

Besides the six fixed dates, we also abstracted the data for the following items for each study-scenario/model: (a) predicted date of every peak in daily / new / active cases or deaths, and (b) predicted date of ‘epidemic control’ or equivalent (with the same study’s criteria or definition of control). Predicted values of main outcomes were recorded for these dates. We also recoded the methods used for as assessment of each statistical model’s validity (or fitness) and their findings.

Two reviewers (FP and MRH) abstracted the estimates from the target studies. For abstraction of the main outcomes’ values at the designated dates, we prioritized our sources and methods as, (1) mention in article text and tables, (2) digitization of article graphs. We used a web-based plot digitizer, “WebPlotDigitizer 4.2” [26]. Our error in digitizing data was less than 5%, as measured using the following formula: error in digitizing = ((digitized value – mentioned value) / (digitized value)), where the mentioned value means the value that was mentioned in study text or tables. We used the reported COVID-19 cases and deaths complied by the Johns Hopkins University [4, 5] for each calendar date, as equal to Iran’s official reported data compiled in WHO situation reports. For developing our graphs, we chose the median scenario/models for cumulative cases from each study in order to demonstrate the main level of the predictions in non-international studies. Only eight non-international studies estimated cumulative deaths, so that outcome could not be used for identification of median scenarios across all studies. In studies with even number of scenario/models, we chose the one (of the middle two) with the higher values of estimates. The same selected median scenario/models for cumulative cases were used to graph the outcomes where the predictions were available. We recorded the text, table number, or the graph number for each study where we extracted every single number or date used in our tables and graphs. “Additional file 2 - Target studies’ abstracted data” includes all the detailed data we abstracted from the studies, as well as detailed findings from the studies’ methods.

## Results

General characteristics of reviewed studies: We found 114 articles, 10 non-peer reviewed reports, and seven web sites that described methods and present results of estimations; a total of 131 results.

We included 18 published articles, two medRxiv preprints, seven web sites of COVID-19 epidemic modeling studies, and two non-peer-reviewed reports; a total of 29 studies. The two non-peer-reviewed reports were in Farsi; Haghdoost [27], and Mashayekhi [28]. One published article was in Farsi; Rahimi Rise [29]. Among the seven included web sites, one estimated the outcomes in Iran (Saberi [21]) and the six others were international studies estimating the outcomes for multiple countries on a periodical basis; DELPHI [10], Gu (YYG) [17], IHME [12], Imperial College [13], LANL [14], and Srivastava [15]. Three studies, DELPHI, Gu (YYG), and Los Alamos did not have a publication, and whatever details about their study methods are available on their study web sites [10, 11, 14]. The three other international studies have at least one publication each (as of 2020-10-29): IHME, peer-review published [30], pre-print [31–33]; Imperial College, peer-review published [34], and Srivastava, pre-print [35]. For each of the six international studies, we mentioned the study web site, study data site, and their publications. For reporting the number of the reviewed component studies, we count each international study as one, rather than creating a hierarchical clustered structure with study publication / study web site at higher level and individual studies at lower level. Appendix Figure 1 shows the PRISMA studies flow diagram.

We report our findings following the ‘preferred reporting items’ mentioned above.

Place: 19 studies included only Iran; the other 13 studies included from 6 to 184 countries. Six studies included subnational level estimates: Haghdoost [27], Moghadami [36], Muniz-Rodriguez [37], Pourghasemi (PLoS ONE) [38], Pourghasemi (IJID) [39], and Zhan [40].

### Epidemic start date and rationale

Twenty studies mentioned the epidemic start date, nine of which used presumed official start date of 2020-02-19. Epidemic start date ranged from 2020-01-02 (Ghaffarzadegan [41]) to 2020-02-20 (Moradi [42], Shen [43]).

Two studies (Moradi [42], Shen [43]) reported their estimates starting from 2020 to 02-20 without mention of the rationale. Ghaffarzadegan reported most of their estimates starting from 2020-01-02, based on unofficial reporting of suspected cases [41]. Haghdoost et al. designated their “Day-zero” as 2020-01-21 [Hijri solar date 1398-11-01], that is 20 days before the presumed official epidemic start date of 2020-02-19 [27]. Many of the predicted outcome values are zero or close to zero in the graphs prior to day 20 of the graphs. However, some of the graphs do seem to show non-zero values for cases or deaths before their day. They maintain that their start date of the epidemic in Iran (2020-01-21) was designated based on “available documentations and epidemiologic analyses”. Mashayekhi et al. did not mention their epidemic start date, and prediction graphs’ time axis showed day zero to 120 or 360 [28]. We made an assumption that their start date was 2020-02-19. Nine studies did not mention the epidemic start date.

### Epidemic (disease) model type and description

Sixteen studies used compartmental models: SEIR or SEIR+ (nine studies), SIR or SIR+ (six studies), SLIR+ (one study). In model acronyms, ‘S’ stands for Susceptible, ‘E’ for Exposed, ‘I’ for Infected, ‘R’ for Removed or Recovered, and ‘L’ is for Latent. In any model with a + sign, there are other components for augmentation of model.

### Statistical model type, description, and equation(s)

Some of the studies did not mentioned enough details about their statistical methods and did not clearly differentiate between the disease model and the statistical model. Statistical methods used included growth models (6 studies), dynamic models (4 studies), Auto-Regressive Integrated Moving Average (ARIMA) (3 studies), regression models (3 studies), and ‘curve fitting and functional analysis’, hyperparametric learning, machine learning, minimization, smoothing model, and time series (each in one study). Few studies provided formal representation (equation) of the model.

### Model assumptions and their verification

None of the reviewed studies did explicitly mention all the assumptions, their verification methods, and results of the verification. Most studies did report some details about their assumptions.

### Model scenarios’ detailed description

There were 77 study-scenarios (epidemic progression scenarios) and 38 study-models (statistical models), resulting in a total of 84 study-model/scenario combinations. Fourteen studies used only one scenario (that practically means no “scenario”). Fifteen other studies used two to 12 scenarios (median 3). Nine studies included policy intervention scenarios with different levels of details mentioned. Eight studies used three levels of interventions, that can be generally formulated as ‘current policies’, ‘more restrictions’, and ‘less restrictions’; Ahmadi [44], Ghaffarzadegan [41], Haghdoost [27], IHME [12], Imperial [13], Mashayekhi [28], Saberi (article) [22], Srivastava [15]. They varied substantially in the level of details provided about what was meant by more (or less) restrictions. Haghdoost also included a baseline scenario of no interventions [27]. Imperial had three additional ‘surged’ scenarios [13]. Mashayekhi and IHME provided detailed-enough description of their more (or less) restrictions [12, 28]. Policy interventions or restrictions included physical social distancing in Haghdoost [27], Mashayekhi [28], Rahimi Rise [29], and IHME [12]. Haghdoost also used patient detection and isolation [27]. Mashayekhi also included level of hygienic precautions practiced by the general population [28]. Rahimi Rise also included alterations in preparations for use of public transit [29]. IHME included use of masks by the general population [12]. Two studies motioned two levels of ‘no-policy’ and ‘actual policies’; Hsiang [45] and Rahimi Rise [29]. Saberi (web site) [21] and Srivastava [15] included under-reporting in official reports. Ghaffarzadegan, Haghdoost, and IHME included seasonality [12, 27, 41]. Tuite and Zhuang included air travel data scenarios early in the epidemic [46, 47]. Ahmadi started with statistical models and reasoned backwards about what intervention scenarios could match each statistical model [16, 44].

Ghaffarzadegan had two policy effect scenarios with different levels of efforts to decrease contact rate as well as three seasonality condition options, that amounted to six total scenarios [41]. Haghdoost had four final scenarios, each with levels of isolation for the infected and suspected patients, as they maintained that “to postpone the heavy wave of the disease, the most effective tool is isolation of patients, in a way that the infected and suspected patients would have the least contact with healthy people”. In the early stages of model building, they modeled “the effects of people’s behaviour change and seasonality on disease transmission”, to show the basic or worst model. Then three intervention scenarios with different levels of isolations were added. The people’s behaviour change and seasonality scenarios end only in the basic or worst scenario with no intervention [27]. Mashayekhi has three scenarios, each with different levels of social [physical] contacts and observation of sanitation cautions. As such, Mashayekhi and IHME were the only studies that considered two modalities of non-pharmacologic interventions [12, 28]. Details of studies’ scenarios are presented in the Appendix.

There were factors other than deaths and / or cases, one or more of which were considered in 11 studies. Eight studies included policy interventions e.g. distancing, quarantine, use of masks by general population; DELPHI [10], Ghaffarzadegan [41], Haghdoost [27], Hsiang [45], Imperial [13], Mashayekhi [28], Rahimi Rise [29], and Thu [48]. Seven studies included asymptomatic cases; DELPHI [10], Ghaffarzadegan [41], Gu (YYG) [17], Mashayekhi [28], Saberi (article) [22], Rahimi Rise [29], and Srivastava [15]. Six studies included under-reporting and / or delays in reporting; DELPHI [10], Ghaffarzadegan [41], Gu (YYG) [17], Saberi (web site) [21], Saberi (article) [22], and Srivastava [15]. Three studies included seasonality; Ghaffarzadegan [41], Haghdoost [27], and IHME [12]. Two studies included testing availability and / or number of tests performed; Ghaffarzadegan [41] and IHME [12]. Two studies included environmental and meteorological variables; Pourghasemi (PLoS ONE) [38] and Pourghasemi (IJID) [39]. One study included seroprevalence, as well as mobility in population samples; IHME [12]. One study included comorbidities, as well as age stratification for mortality; Imperial [13]. One study included changes to domestic COVID-19-testing regimes, such as case definitions or testing methodology; Hsiang [45]. The following items were not incorporated in any of the scenarios of the included studies: potential vaccine(s), potential pharmacological treatments, changes in cause of death definition, possibility of reinfection, and possibility of mutations or any change in virulence.

### Validation process and findings

Eighteen studies provided one or more measures and results for model validation: Root Mean Squared Error (RMSE) or Mean Squared Error (MSE) (9 studies), Mean Absolute Percentage Error (MAPE) (4 studies), Mean Absolute Error (MAE) (3 studies), Ratio Error (RE) (2 studies), R square (2 studies), Area Under Curve (AUC) (2 studies), Akaike Information Criterion (AIC) (2 studies), and Root Mean Squared Relative Error (RMSRE) (1 study).

### List and sources of model parameters and input data

Twenty-three studies used deaths and / or cases as input data, source of which included MOHME official reports (10 studies), Johns Hopkins University [4, 5] (6 studies), source not mentioned (6 studies), and Worldometers web site [49] (4 studies). Cases data were used in 23 studies and deaths data in 18 studies. Eleven studies used additional input variables other than deaths and or cases; eight studies used Non-Pharmaceutical Intervention (NPI) variables and three studies used testing data. List and sources of model parameters are available in the Supplementary electronic material (“Studies’ Methods” tab). In Haghdoost’s study, for number of deaths and cases to start with, assumptions were made that on day-zero, there had been 1080 persons exposed to the virus In Iran (including 75 in Tehran), from which 90 persons had become infected in Iran (including 5 in Tehran) [27]. Four studies did not report using number of confirmed cases or confirmed deaths as model input; Tuite [46], Zhuang [47], Haghdoost [27], and Mashayekhi [28]. Among other studies, Ghaffarzadegan used other sources of data, including unofficial reports for number of cases and death and number of performed tests [41].

### Model outputs preferably with uncertainty intervals for scenarios

Primary outcomes: The most frequent type of main outcome was cumulative cases only (seven studies). Other studies reported a combination of cumulative or daily deaths or cases. Mashayekhi [28] reported estimates of symptomatic and symptomatic cases separately, and Saberi (article) [22] reported total number of confirmed and suspected cases together. Thirteen studies provided confidence intervals for the primary outcomes.

Forms of primary outcomes: The intended outcomes and the terminology used in the included studies for the same outcomes, varied across the studies. For daily cases, two distinct groups could be recognized: daily incident cases, and daily prevalent cases. Our designation of daily incident cases included “new cases” reported daily by MOHME (via [4, 5]), “new cases” predicted by Haghdoost [27], and “daily cases” by Zareie [50]. Our designation of daily prevalent cases included “current cases” by Ghaffarzadegan [41], “maximum number of cases per day” by Haghdoost [27], “daily cases” by Mashayekhi [28], and “daily active cases” by Saberi (web site) [21]. Active cases are the difference between total cumulative cases with cumulative number of deceased and recovered cases. Saberi (article) reported estimated sum of daily confirmed and suspected cases [22].

Other outcomes: Four studies reported outcomes other than deaths and / or cases. They reported different combinations of hospitalization demand estimates (all beds, intensive care unit beds, invasive ventilators); DELPHI [10], Haghdoost [27], IHME [12], and Imperial [13].

Date range of estimates for deaths and / or cases: Start dates of outputs ranged from 2019-12–31 (Ghaffarzadegan [41]) to 2020-09-19 (Srivastava [15]). End dates of outputs ranged from 2020-02-24 (Zhuang [47]) to 2021-02-02 (Saberi (web site) [21]). Outputs duration ranged from 11 days (Muniz-Rodriguez [37]) to 364 days (IHME [12]).

R0 estimation results: Eleven studies reported estimated Reproductive Number values, ranging from 0.69 (95% CI 0.68–0.70) on 15 April 2020 (after control measures that took place) by Saberi (article) [22], to 7.24 at the beginning of the epidemic by Haghdoost [27].

Twenty-one studies mentioned their study limitations, among which 12 studies really described the limitations, and the nine others touched the limitations very minimally.

Increase in percent population using masks from the current situation (i.e. current scenario) to 95% (i.e. best scenario, 95% mask usage in public in every location) might prevent 26,790 additional deaths (95% confidence interval 19,925–35,208) by the end of year 2020 (IHME [12]).

Table 1 summarizes the findings regarding the methodology used in the reviewed studies. The six fixed dates, the ends of months one to six, are used and shown in Table 2. Table 2 shows the estimates of cumulative deaths. Table 3 summarizes the outcomes at the end of month two (2020-04-19) and month four (2020-06-20) after the official epidemic start date, and the latest dates available in 2020 and 2021. Estimates of cumulative cases, daily deaths and daily cases are demonstrated in Appendix Tables 1, 2, and 3 respectively. Appendix Table 4 demonstrates predictions of peak dates and values of outcomes, and Appendix Table 5 shows predictions of epidemic control dates and values of outcomes.

**Table 1:** Reported items of methodology of the reviewed studies

Study first author	Ahmadi [44]		Al-Qaness [51]	Ayyoubzadeh [52]	DELPHI [10]	Ghaffarzadegan [41]	Gu (YYG) [17]	Haghdoost [27]
Situation of study	Published paper		Published paper	Published paper	Web site	Published paper	Web site	Full report (Farsi)
Epidemic start date	20-02-19		20-01-22	20-02-11	N/M ^a^	20-01-02	20-01-26	20-01-21
Inputs: population	N/M ^a^		N/M ^a^	N/M ^a^	Yes	Yes	Yes	Yes
Inputs: cases	Yes		Yes	Yes	Yes	Yes	No	No
Inputs: cases (source)	MOHME ^b^ official reports		World Health Organization	Worldometers website ^c^	Johns Hopkins University ^d^	MOHME ^b^ official reports; unofficial reports	Johns Hopkins University ^d^	N/A ^e^
Inputs: deaths	Yes		No	No	Yes	Yes	Yes	No
Inputs: deaths (source)	MOHME ^b^ official reports		N/A ^e^	N/A ^e^	Johns Hopkins University ^d^	MOHME ^b^ official reports; unofficial reports	Johns Hopkins University ^d^	N/A ^e^
Other input data	Number of cured [recovered] cases		N/A ^e^	N/A ^e^	Nonpharmaceutical interventions	Number of tests, Detected infected travelers, Travel data	Case and hospitalization data ^f^	Post-infection isolated persons, Hospitalized cases, Infected cases recovered without isolation or hospitalization
Output date range (number of days)	20-02-19 to 20-04-03 (45 days)		20-01-22 to 20-04-07 (77 days)	20-02-11 to 20-03-18 (37 days)	20-06-01 to 20-07-15 (45 days)	19-12-31 to 20-06-30 (183 days)	20-01-26 to 20-11-01 (281 days)	20-01-21 to 20-05-20 (121 days)
Place	Iran		4 countries	Iran	148 countries	Iran	70 countries	Iran and Tehran capital city
Compartmental model ^g^	SIR ^g^		None	None	SEIR+ ^g, h^	SEIR+ ^g^	SEIR ^g^	SEIR+ ^g^
Statistical method: name	3 growth models ^i^		6 time-series models ^j^	2 Models ^k^	Regression trees	Dynamic simulation model	Machine learning	Dynamic model
R0 estimation results	1.75		None	None	None	2.72 (before starting the interventions)	4 estimates ^k^	3 estimates ^l^
Scenarios /models: number	3 ^m^		1	1	1	6 ^n^	1	4 ^o^
Other factors	No		No	No	Yes. Asymptomatic cases, under-reporting	Yes ^p^	Yes. Asymptomatic cases, under-reporting	Yes ^q^
Primary outcomes	Cumulative deaths, Cumulative cases		Cumulative cases	Normalized Daily cases	Cumulative and daily deaths and cases	Cumulative deaths, Cumulative cases, Current cases	Cumulative and daily deaths and cases, Daily prevalent cases	Cumulative and daily deaths and cases, Daily prevalent cases
Primary outcomes interval estimates	No		No	No	No	No	Yes	No
Other outcomes	None		None	None	Active, Active hospitalized, Cumulative hospitalized, Active ventilated	None	Reproduction Number	Needed hospital beds, ICU beds
Other outcomes interval estimates	N/A ^e^		N/A ^e^	N/A ^e^	No	N/A ^e^	Yes	No
Model validation	No		Yes ^r^	Yes ^s^	No ^t^	Yes ^u^	Yes ^v^	No
Study limitations mentioned	Yes		Yes	Yes	Yes	Yes	Yes	No
Study limitations described	Yes		No	No	Yes	No	Yes	No
Study first author	Hsiang [45]		IHME [12]	Imperial [13]	LANL [14]	Mashayekhi [28]	Moftakhar [53]	Moghadami [36]
Situation of study	Published paper		Web site [12] and published paper [30]	Web site [13] and published paper [34]	Web site	Summary report (Farsi)	Published paper	medRxiv preprint
Epidemic start date	N/M ^aa^		N/M ^aa^	20-01-03	N/M ^aa^	20-02-19 [?]	20-02-19	20-02-19
Inputs: population	Yes		Yes	Yes	Yes	Yes	No	No
Inputs: cases	Yes		Yes	Yes	Yes	No	Yes	Yes
Inputs: cases (source)	Wikipedia ^bb^		Johns Hopkins University ^cc^	Johns Hopkins University ^cc^	Johns Hopkins University ^cc^	N/A ^dd^	MOHME ^ee^ and Johns Hopkins ^cc^	MOHME ^ee^
Inputs: deaths	Yes		Yes	Yes	Yes	No	No	Yes
Inputs: deaths (source)	Wikipedia ^bb^		Johns Hopkins University ^cc^	Johns Hopkins University ^cc^	Johns Hopkins University ^cc^	N/A ^dd^	N/A ^dd^	MOHME ^ee^
Other input data	3 variables ^ff^		4 variables ^gg^	5 variables ^hh^	N/M ^aa^	N/M ^aa^	N/M ^aa^	None
Output date range (number of days)	~ 20-02-28 to 20-04-06 (~ 39 days)		20-02-04 to 21-02-01 (364 days)	20-01-06 to 20-11-24 (324 days)	20-03-14 to 20-11-07 (239 days)	N/M ^aa^ (360 days)	20-03-21 to 20-04-20 (31 days)	20-03-21 to 20-04-20 (31 days)
Place	6 countries		165 countries	164 countries	157 countries	Iran	Iran	Iran and top 5 provinces
Compartmental model ^ii^	SIR+ ^ii^		SEIR ^ii^	SIR, SEIR, SEIR+ ^ii^	SEIR+ ^ii^	SLIR+ ^ii^	None	None
Statistical method: name	Multiple regression		Curve fitting (backcating) functional analysis (forecasting)	Regression trees	Dynamic growth parameter modeling	Dynamic model	Autoregressive Integrated Moving Average (ARIMA)	Exponential smoothing model
R0 estimation results	Not used		N/M ^aa^	N/M ^aa^	N/M ^aa^	Not used	Not used	Not used
Scenarios /models: number	2 ^jj^		3 ^kk^	6 ^ll^	1	3 ^mm^	1	1
Other factors	Yes. Under-reporting.		Yes ^nn^	Yes. Under-reporting.	Yes. Under-reporting.	Yes ^oo^	No	No
Primary outcomes	Cumulative cases		Cumulative and daily deaths and cases	Cumulative and daily deaths and cases	Cumulative and daily deaths and cases	Cumulative and daily deaths, Daily symptomatic and asymptomatic cases	Daily cases	Cumulative deaths, cases, recovered cases
Primary outcomes interval estimates	Yes		Yes	Yes	Yes	No	Yes	Yes
Other outcomes	None		Yes ^pp^	Yes ^qq^	None	None	None	None
Other outcomes interval estimates	N/A ^dd^		Yes	Yes	N/A ^dd^	N/A ^dd^	N/A ^dd^	N/A ^dd^
Model validation	(?)		Yes ^rr^	Yes ^ss^	Yes ^tt^	No	Yes ^uu^	Yes ^vv^
Study limitations mentioned	Yes		Yes	No	No	Yes	Yes	No
Study limitations described	Yes		Yes	No	No	No	Yes	No
Study first author	Moradi [42]	Muniz-Rodriguez [37]	Pourghasemi (PLoS ONE) [38]	Pourghasemi (IJID) [39]	Rafieenasab [54]	Rahimi Rise [29]	Saberi (web site) [21]	Saberi (paper) [22]
Situation of study	Published paper	Published paper	Published paper	Published paper	Published paper	Published paper (Farsi)	Web site [21]	Published paper
Epidemic start date	20-02-20	20-02-19	20-02-25 [?]	20-02-25 [?]	20-02-19	20-02-01	20-02-19	20-02-19
Inputs: population	No	N/M ^aaa^	Yes	Yes	N/M ^aaa^	Yes	N/M ^aaa^	Yes
Inputs: cases	No	Yes	Yes	Yes	Yes	Yes	Yes	Yes
Inputs: cases (source)	N/A ^bbb^	MOHME ^ccc^ official reports	MOHME ^ccc^ official reports	MOHME ^ccc^ official reports	MOHME ^ccc^ official reports	Worldometers website ^ddd^	MOHME ^ccc^ official reports, WHO, Worldometers ^ddd^	MOHME ^ccc^ official reports, WHO
Inputs: deaths	Yes	No	Yes	Yes	No	Yes	Yes	Yes
Inputs: deaths (source)	MOHME ^ccc^ official reports	N/A ^bbb^	MOHME ^ccc^ official reports	MOHME ^ccc^ official reports	N/A ^bbb^	Worldometers website ^ddd^	MOHME ^ccc^ official reports, WHO, Worldometers ^ddd^	MOHME ^ccc^ official reports, WHO, Worldometers ^ddd^
Other input data	None	Travel data	Environmental and meteorological conditions	Environmental and meteorological conditions	None	Public transportation variables	None	None
Output date range (number of days)	20-02-20 to 20-03-26 (36 days)	20-02-19 to 20-02-29 (11 days)	~ 20-02-25 to ~ 20-06-10 (~ 107 days) ^eee^	~ 20-02-25 to ~ 20-06-20 (~ 117 days) ^fff^	20-02-19 to 20-06-07 (110 days)	20-02-01 to 20-08-01 (183 days)	20-02-19 to 21-02-02 (350 days)	~ 20-03-19 to 20-10-26 (~ 222 days)
Place	Iran	Iran and 2 multi-province regions	Iran and Fars Province	Iran, 31 Provinces of Iran, World	Iran	Iran	Iran	Iran
Compartmental model ^ggg^	None	None	None	None	SIR+ ^ggg, hhh^	SEIR ^ggg^	SIR ^ggg^	SEIR+ ^ggg, iii^
Statistical method: name	Calculating number of cases based on different assumptions for case fatality rate (CFR)	Generalized growth mode; Based on the calculation of the epidemic doubling times	Autoregressive Integrated Moving Average (ARIMA) and polynomial regression	Fourth-degree polynomial regression	3-steps model based on the SIR model	Dynamic model	Classical SIR^ggg^ mathematical model with homogenous mixing assumption	Ordinary least squares minimization
R0 estimation results	Not used	Two methods: 3.6 and 3.58	Not used	Not used	2.8–3.3 (range)	Not used	2.37 (for the last 7 days before 20-03-21)	1.73 (20-03-01) and 0.69 (2004-15) ^jjj^
Scenarios /models: number	4 ^kkk^	2 ^lll^	1	1	1	2	12 ^mmm^	3 ^nnn^
Other factors	No	No	No	No	No	Yes. Asymptomatic cases	Yes ^ooo^	Yes ^ppp^
Primary outcomes	Cumulative cases	Daily cases	Cumulative and daily deaths and cases ^qqq^	Cumulative and daily deaths and cases ^rrr^	Cumulative and daily deaths, Daily cases	Daily deaths and cases	Cumulative cases, Daily active cases	Fractions of national population estimated to be confirmed and suspected cases ^sss^
Primary outcomes interval estimates	No	Yes	No	No	No	No	No	Yes
Other outcomes	Case Fatality Rate	None	None	None	None	None	None	Intensive Care Unit beds needed
Other outcomes interval estimates	No	N/A ^bbb^	N/A ^bbb^	N/A ^bbb^	N/A ^bbb^	N/A ^bbb^	N/A ^bbb^	Yes
Model validation	No	No	Yes ^ttt^	Yes ^uuu^	No	Yes ^vvv^	No	Yes ^www^
Study limitations mentioned	Yes	Yes	No	Yes	No	No	Yes	Yes
Study limitations described	No	Yes	No	No	No	No	No	Yes
Study first author	Shen [43]		Singh [55]	Srivastava [15]	Thu [48]	Tuite [46]	Zhan [40]	Zhuang [47]
Situation of study	Published paper		Published paper	Web site [15] and preprint [35]	Published paper	Published paper	Published paper	Published paper
Epidemic start date	20-02-20		N/M ^aaaa^	N/M ^aaaa^	N/M ^aaaa^	N/M ^aaaa^	20-02-19	N/M ^aaaa^
Inputs: population	No		No	N/M ^aaaa^	No	N/M ^aaaa^	N/M ^aaaa^	Yes
Inputs: cases	Yes		Yes	Yes	Yes	No	Yes	No
Inputs: cases (source)	“WIND DATA” ^bbbb^		Worldometers ^cccc^	Johns Hopkins University ^dddd^	WHO	N/A ^bbbb^	MOHME ^eeee^ official reports	N/A ^ffff^
Inputs: Deaths	No		No	Yes	Yes	No	Yes	No
Inputs: Deaths (source)	N/M ^aaaa^		N/M ^aaaa^	Johns Hopkins University ^dddd^	WHO	N/A ^ffff^	WHO	N/A ^ffff^
Other input data	None		None	None	Social distancing	Exported cases from Iran to other countries; Travel data	COVID-19 spreading profiles of 367 cities in China	Exported cases from Iran to other countries, Travel data
Output date range (number of days)	20-02-20 to 20-04-20 (61 days)		20-04-24 to 20-07-07 (75 days)	20-09-19 to 20-12-19 (every 7th day, 14 dates, 92 days duration)	20-03-30 to 20-05-02 (34 days)	20-01-01 to N/M ^aaaa^	20-02-22 to 20-06-24 (124 days)	20-02-01 to 20-02-24 (24 days)
Place	9 countries and 11 provinces / municipalities in China		15 countries	184 countries	10 countries	Iran	Iran and 12 provinces	Iran
Compartmental model ^gggg^	None		None	SIR+ ^gggg, hhhh^	None	None	SEIR+ ^gggg^	None
Statistical method: name	Logistic growth		Autoregressive Integrated Moving Average (ARIMA)	Hyper-parametric learning	Linear growth rates ^iiii^	N/M ^aaaa^	Data-driven prediction algorithm ^kkkk^	Binomial distributed likelihood framework
R0 estimation results	Not used		Not used	1.44 (20-03-21), 1.46 (20-03-28)	Not used	Not used	Not used	Not used
Scenarios /models: number	1		1	3 ^llll^	1	6 ^mmmm^	1	5 ^nnnn^
Other factors	No		No	Asymptomatic cases, under-reporting	No	No	No	No
Primary outcomes	Cumulative cases		Cumulative cases	Cumulative deaths and cases	Daily cases	Cumulative cases	Cumulative and daily cases	Cumulative cases
Primary outcomes interval estimates	No		Yes	No	No	Yes	Yes	Yes
Other outcomes	None		None	None	None	None	None	None
Other outcomes interval estimates	N/A ^ffff^		N/A ^ffff^	N/A ^ffff^	N/A ^ffff^	N/A ^ffff^	N/A ^ffff^	N/A ^ffff^
Model validation	Yes ^oooo^		Yes ^pppp^	Yes ^qqqq^	No	No	Yes ^jjjj^	No
Study limitations mentioned	Yes		Yes	Yes	Yes	No	Yes	Yes
Study limitations described	No		Yes	Yes	Yes	No	Yes	No

^a^ N/M: Not Mentioned

^b^ MOHME: Ministry of Health and Medical Education, Iran

^c^ Worldometers Coronavirus [49]

^d^ Johns Hopkins University, Coronavirus Resource Center ([4, 5])

^e^ N/A: Not applicable

^f^ “We do not use case-related data in our modeling. We do look at case and hospitalization data to help determine the bounds for our search grid, as changes in cases lead changes in deaths.” Gu (YYG) [17]

^g^ Compartmental models: S: Susceptible, E: Exposed, I: Infected, R: Removed or Recovered, L: Latent. In any model with a + sign, there are other components for augmentation of model

^h^ DELPHI model: The model underlying the predictions is DELPHI (Differential Equations Leads to Predictions of Hospitalizations and Infections), that is based on SEIR with augmentations for under-detection and governmental response. DELPHI [10]

^i^ Three growth models: M1: Gompertz Differential Equation, M2: Von Bertalanffy differential growth equation, and M3: Cubic polynomial least squared errors

^j^ Six time-series models: (1) Adaptive Neuro-Fuzzy Inference System (ANFIS) enhanced with Genetic Algorithm (GA), (2) Original Adaptive Neuro-Fuzzy Inference System (ANFIS), (3) Particle Swarm Optimizer (PSO), (4) Artificial Bee Colony (ABC), (5) hybridized of Flower Pollination Algorithm and SALP Swarm Algorithm (SSAFPA), (6) Sine-Cosine Algorithm (SCA)

^h^ Two Models: Linear Regression, Long Short-Term Memory (LSTM)

^k^ Four estimates: Initial R0 = 2.65. Reopen R = 1.17. Current R = 1.2. Post-mitigation R = 0.90

^l^ Three estimates: 7.24 (at the beginning). 2.58 (after interventions). 1.82 (conditional to isolation of 50% within 3 days)

^m^ Three scenarios based on 3 growth models: S1: Gompertz Differential Equation, S2: Von Bertalanffy differential growth equation, and S3: Cubic polynomial least squared errors

^n^ Six scenarios based on combination of two factors: Seasonality (S), and Policy interventions (P). (1) S1P1: Seasonality conditions 1 (no effect or status quo) and Policy effect 1 (status quo contact rate). Estimates for 2020-03-19, the end of first month after the epidemic start date, are equal across the six scenarios. (2) S1P2: Seasonality conditions 1 (no effect or status quo) and Policy effect 2 (aggressive efforts to decrease contact rate by half of what it would be otherwise). (3) S2P1: Seasonality conditions 2 (moderate effect; infectivity of the virus decreases linearly from April 1st and halves by June 1st, then stays the same for the rest of the simulation) and Policy effect 1 (status quo contact rate). (4) S2P2: Seasonality conditions 2 (moderate effect; infectivity of the virus decreases linearly from April 1st and halves by June 1st, then stays the same for the rest of the simulation) and Policy effect 2 (aggressive efforts to decrease contact rate by half of what it would be otherwise). (5) S3P1: Seasonality conditions 3 (very strong mitigating effect; infectivity of the virus decreases from April 1st to a quarter of its base value by June 1st, then stays the same for the rest of the simulation) and Policy effect 1 (status quo contact rate). (6) S3P2: Seasonality conditions 3 (very strong mitigating effect; infectivity of the virus decreases from April 1st to a quarter of its base value by June 1st, then stays the same for the rest of the simulation) and Policy effect 2 (aggressive efforts to decrease contact rate by half of what it would be otherwise)

^o^ Four scenarios: S0: Basic scenario (no intervention), only 10% isolation. S1: Worst scenario, minimum (25%) isolation. S2: Medium scenario, medium (32%) isolation. S3: Best scenario, maximum (40%) isolation

^p^ Seven other factors included: Asymptomatic cases, Under-reporting / Completeness of reporting cases and deaths to MOHME, Delays in reporting cases and deaths to MOHME, Testing availability, Number of tests performed, Social distancing / Quarantine interventions, Seasonality

^q^ Two other factors included: Seasonality, Social distancing / Quarantine interventions

^r^ Root Mean Squared Error (RMSE), Mean Absolute Error (MAE), Mean Absolute Percentage Error (MAPE), Root Mean Squared Relative Error (RMSRE), and Coefficient of Determination (R square)

^s^ Root Mean Squared Error (RMSE)

^t^ Friedman [31] assessed predictive performance of international COVID-19 mortality forecasting models, using median absolute percent error (MAPE) and Median absolute errors (MAE)

^u^ Root Mean Squared Error (RMSE)

^v^ Mean Square Error (MSE), Mean Absolute Error (MAE), and Ratio Error (RE). Did not mention the results

^aa^ N/M: Not Mentioned

^bb^ Wikipedia. COVID-19 pandemic in Iran [56]

^cc^ Johns Hopkins University, Coronavirus Resource Center ([4, 5])

^dd^ N/A: Not Applicable

^ee^ MOHME: Ministry of Health and Medical Education, Iran

^ff^ Four variables: Cumulative recoveries, Active cases, Any changes to domestic COVID-19-testing regimes, such as case definitions or testing methodology, and Non-pharmaceutical interventions

^gg^ Three variables: Mobility, Testing, and Seroprevalence (the latter for 41 locations)

^hh^ Five variables: Interventions, Social contacts, Comorbidities, Hospital bed capacity, Intensive Care Unit bed capacity

^ii^ Compartmental models: S: Susceptible, E: Exposed, I: Infected, R: Removed or Recovered, L: Latent. In any model with a + sign, there are other components for augmentation of model

^jj^ Two scenarios: ‘No-policy scenario’ and ‘Actual policies’

^kk^ Three scenarios: S1 Best (Masks): ‘Universal Masks’ scenario reflects 95% mask usage in public in every location. S2 Reference (Current): ‘Current projection’ scenario assumes social distancing mandates are re-imposed for 6 weeks whenever daily deaths reach 8 per million (0.8 per 100,000). S3 Worse (Easing): ‘Mandates easing’ scenario reflects continued easing of social distancing mandates, and mandates are not re-imposed

^ll^ Six scenarios: S1: Additional 50% Reduction. S2: Maintain Status Quo. S3: Relax Interventions 50%. S4: Surged Additional 50% Reduction. S5: Surged Maintain Status Quo. S6: Surged Relax Interventions 50%

^mm^ S1: Ideal scenario, serious distancing. People reduce their social [physical] contacts to 20% of regular level, voluntarily or on a forced basis, after number of cases and deaths have increased, plus close observation of sanitation cautions, so that transmission rate reduces by 65%. S2: Medium scenario, not serious distancing. People reduce their social [physical] contacts only to 20% of regular level, voluntarily, after number of cases and deaths have increased, and other settings are like scenario 1. S3: Worst scenario. People reduce their social [physical] contacts only to 50% of regular level, voluntarily, after number of cases and deaths have increased, plus inadequate observation of sanitation cautions, so that transmission rate reduces only by 40% (instead of 55%), and 60% of people do not observe the sanitation cautions

^nn^ Five other factors included: Asymptomatic cases, Mobility, Testing, Seroprevalence, Seasonality

^oo^ Two other factors included: Asymptomatic cases, Social distancing / Quarantine interventions

^pp^ Six other outcomes: All beds needed, Intensive Care Unit beds needed, Invasive ventilators needed, Tests, Mobility, Seroprevalence

^qq^ Five other outcomes: Hospital demand, Hospital incidence, Intensive Care Unit demand, Intensive Care Unit incidence, Rt (Effective Reproduction Number)

^rr^ IHME web site [12] refers to Friedman [31], who assessed predictive performance of international COVID-19 mortality forecasting models, using median absolute percent error (MAPE) and Median absolute errors (MAE)

^ss^ Mean Absolute Percentage Error (MAPE)

^tt^ They validated the model “by looking at the coverage of the forecasts, i.e. the proportion of times that the number of confirmed cases/deaths fell within a specified lower and upper bound, X min and X max. Coverage plots can help visualize how well the model is doing”

^uu^ Graphical residual assessment of the model

^vv^ Mean Squared Error (MSE), Root Mean Squared Error (RMSE), Mean Absolute Error (MEA), Mean Absolute Percentage Error (MAPE), Akaike Information Criterion (AIC)

^aaa^ N/M: Not Mentioned

^bbb^ N/A: Not Applicable

^ccc^ MOHME: Ministry of Health and Medical Education, Iran

^ddd^ Worldometers Coronavirus [49]

^eee^ Start and end dates mentioned in manuscript text, mentioned in title of their Fig. 14, and shown within their Fig. 14 do not seem to be congruent

^fff^ Start and end dates mentioned in manuscript text, mentioned in title of their Fig. 15, and shown within their Fig. 15 do not seem to be congruent

^ggg^ Compartmental models: S: Susceptible, E: Exposed, I: Infected, R: Removed or Recovered, L: Latent. In any model with a + sign, there are other components for augmentation of model

^hhh^ SIR with exact and approximated solutions, extrapolation based on least squares model with three functions

^iii^ SEIR+ Distinguishing between fatal and recovered cases combined with an estimate of the percentage of symptomatic cases using delay-adjusted Case Fatality Rate

^jjj^ Estimated effective reproduction number that ranged from 0.66 to 1.73 between February and April 2020, with a median of 1.16. Estimated a reduction in the effective reproduction number during this period, from 1.73 (95% CI 1.60–1.87) on 1 March 2020 to 0.69 (95% CI 0.68–0.70) on 15 April 2020, due to various non-pharmaceutical interventions

^kkk^ Four scenarios based on different values of Case Fatality Rate. S1: 0.3%, S2: 0.5%, S3: 1%, and S4: 2%

^lll^ Based on two different methods to estimate R0

^mmm^ (1) S1P10: Scenario 1 (Best scenario, based on official reports with correction factor of 1) with 10 million susceptible population. (2) 12 scenarios based on combination of three options for number of cases and deaths to start with, and four options for the susceptible population size. (1) S1P10: Scenario 1 (Best scenario, based on official reports with correction factor of 1) with 10 million susceptible population. (2) S1P30:Scenario 1 (Best scenario, based on official reports with correction factor of 1) with 30 million susceptible population. (3) S1P50: Scenario 1 (Best scenario, based on official reports with correction factor of 1) with 50 million susceptible population. (4) S1P80: Scenario 1 (Best scenario, based on official reports with correction factor of 1) with 80 million susceptible population. (5) S2P10: Scenario 2 (Medium scenario, based on official reports with correction factor of 5 (after Dr. Rick Brennan, Director of Emergency Operations, World Health Organization [57]) with 10 million susceptible population. (6) S2P30: Scenario 2 (Medium scenario, based on official reports with correction factor of 5 (after Dr. Rick Brennan, Director of Emergency Operations, World Health Organization [57]) with 30 million susceptible population. (7) S2P50: Scenario 2 (Medium scenario, based on official reports with correction factor of 5 (after Dr. Rick Brennan, Director of Emergency Operations, World Health Organization [57]) with 50 million susceptible population. (8) S2P80: Scenario 2 (Medium scenario, based on official reports with correction factor of 5 (after Dr. Rick Brennan, Director of Emergency Operations, World Health Organization [57]) with 80 million susceptible population. (9) S3P10: Scenario 3 (Worst scenario, based on official reports with correction factor of 10 (after Russell [58]) with 80 million susceptible population. (10) S3P30: Scenario 3 (Worst scenario, based on official reports with correction factor of 10 (after Russell [58]) with 30 million susceptible population. (11) S3P50: Scenario 3 (Worst scenario, based on official reports with correction factor of 10 (after Russell [58]) with 50 million susceptible population. (12) S3P80: Scenario 3 (Worst scenario, based on official reports with correction factor of 10 (after Russell [58]) with 10 million susceptible population

^nnn^ Three scenarios: (1) maintaining the same level of control measures as of 12 April 2020, (2) reinforcing the control measures to increase physical distancing by a 20% increase in the reproduction number, and (3) partial lifting the restrictions to ease physical distancing by a 20% decrease in the reproduction number

^ooo^ Completeness of reporting cases and deaths to MOHME

^ppp^ Accounted for the under-reporting of the number of infected cases using delay-adjusted case fatality ratio (CFR) approach

^qqq^ Cumulative deaths and cases (for Iran and Fars Province), Daily deaths and cases (for Fars Province)

^rrr^ Cumulative deaths and cases (for Iran and World), Daily or cumulative cases in 30 days after the first day of infected cases in the 31 Iranian provinces)

^sss^ We transformed their reported fractions of national population estimated to be confirmed and suspected cases to numbers of people estimated to be confirmed and suspected cases, using a total national population of 84,297,880 (used by IHME [12])

^ttt^ Area Under Curve (AUC)

^uuu^ Area Under Curve (AUC)

^vvv^ Root Mean Squared Error (RMSE)

^www^ Root Mean Squared Error (RMSE)

^aaaa^ N/M: Not mentioned

^bbbb^ Mentioned: “WIND DATA, a leading financial data services provider in China”

^cccc^ Worldometers Coronavirus [49].

^dddd^ Johns Hopkins University, Coronavirus Resource Center ([4, 5])

^eeee^ MOHME: Ministry of Health and Medical Education, Iran

^ffff^ N/A: Not Applicable

^gggg^ SEIR+ Distinguishing between fatal and recovered cases combined with an estimate of the percentage of symptomatic cases using delay-adjusted Case Fatality Rate

^hhhh^ SI-kJ alpha model: S: Susceptible. I: Infected. k: k sub-states of infection. J: J is a hyperparameter introduced for a smoothing effect to deal with noisy data. Alpha: an additional hyperparameter to minimizes the Root Mean Squared Error

^iiii^ They have not named their method. It could be names as linear growth rates, according to their Eq. (1) and Eq. (2)

j Another study by Zhan and colleagues was cited for validity of their models

^kkkk^ A data-driven prediction algorithm to find the most resembling growth curve from the historical profiles in China

^llll^ Three scenarios: Current, Released, Restricted, each with 6 levels of putative under-ascertainment parameter

^mmmm^ Six scenarios based on six sets of international travel destinations

^nnnn^ Five scenarios based on selected combinations of (1) Effective catchment population, (2) Detection window 10 or 8 days, and (3) 90% or 70% load factors

^oooo^ R Square

^pppp^ Akaike Information Criterion (AIC)

^qqqq^ Root Mean Squared Error (RMSE)

**Table 2:** Predictions of cumulative deaths for the end of months one to six after the official epidemic start date (2020-02-19) and the latest date available in 202

	Date1^a^	20-03-19	20-04-19	20-05-20	20-06-20	20-07-21	20-08-21	Latest date
	Date 2 ^b^	98-12-29	99-01-31	99-02-31	99-03-31	99-04-31	99-05-31	in 2020 ^c^
- First Author, Outcome	S/M ^d^	Value	Value	Value	Value	Value	Value	Value
- MOHME official via ([4, 5])								
Cumulative deaths	N/A ^e^	1,284	5,118	7,183	9,507	14,634	20,376	30,712
- Ahmadi [44]								
Cumulative deaths	M1 ^f^	1,264	··	··	··	··	··	··
Cumulative deaths	M2 ^g^	1,322	··	··	··	··	··	··
Cumulative deaths	M3 ^h^	1,263	··	··	··	··	··	··
- DELPHI [10]								
Total detected deaths	S1^i^	··	··	··	8,426	··	··	··
- Ghaffarzadegan [41]								
Cumulative deaths	S1P1 ^j^	15,317	44,078	70,462	95,658	··	··	··
Cumulative deaths	S1P2 ^k^	15,317	41,702	52,937	66,549	··	··	··
Cumulative deaths	S2P1 ^l^	15,317	44,078	68,383	85,262	··	··	··
Cumulative deaths	S2P2 ^m^	15,317	41,702	52,937	60,015	··	··	··
Cumulative deaths	S3P1 ^n^	15,317	44,078	68,383	80,213	··	··	··
Cumulative deaths	S3P2 ^o^	15,317	41,702	52,937	57,341	··	··	··
- Gu (YYG) [17]								
Cumulative deaths, mean	S1^i^	··	··	··	··	··	31,955	··
Cumulative deaths, lower	S1^i^	··	··	··	··	··	29,231	··
Cumulative deaths, upper	S1^i^	··	··	··	··	··	36,014	··
- Haghdoost [27]								
Cumulative deaths	S0 ^p^	··	··	30,700	··	··	··	··
Cumulative deaths	S1 ^q^	3,824	9,107	13,450	··	··	··	··
Cumulative deaths	S2 ^r^	2,796	6,231	8,632	··	··	··	··
Cumulative deaths	S3 ^s^	··	··	6,030	··	··	··	··
- IHME [12]								
Cumulative deaths, mean ^t^	S1 ^u^	1,215	5,150	7,183	9,495	14,642	20,369	44,087
Cumulative deaths, lower ^t^	S1 ^u^	1,215	5,150	7,183	9,495	14,642	20,369	38,031
Cumulative deaths, upper ^t^	S1 ^u^	1,215	5,150	7,183	9,495	14,642	20,369	51,027
Cumulative deaths, mean ^t^	S2 ^v^	1,215	5,150	7,183	9,495	14,642	20,369	67,186
Cumulative deaths, lower ^t^	S2 ^v^	1,215	5,150	7,183	9,495	14,642	20,369	57,913
Cumulative deaths, upper ^t^	S2 ^v^	1,215	5,150	7,183	9,495	14,642	20,369	72,170
Cumulative deaths, mean ^t^	S3 ^w^	1,215	5,150	7,183	9,495	14,642	20,369	70,877
Cumulative deaths, lower ^t^	S3 ^w^	1,215	5,150	7,183	9,495	14,642	20,369	57,956
Cumulative deaths, upper ^t^	S3 ^w^	1,215	5,150	7,183	9,495	14,642	20,369	86,235
- Imperial [13]								
Cumulative deaths, mean	S1 ^x^	763	3,743	5,276	7,303	11,537	16,538	27,195
Cumulative deaths, lower	S1 ^x^	434	2,095	3,067	4,203	6,537	9,895	17,638
Cumulative deaths, upper	S1 ^x^	1,254	6,096	8,462	11,620	17,058	23,543	36,103
Cumulative deaths, mean	S2 ^y^	763	3,743	5,276	7,303	11,537	16,538	32,372
Cumulative deaths, lower	S2 ^y^	434	2,095	3,067	4,203	6,537	9,895	19,989
Cumulative deaths, upper	S2 ^y^	1,254	6,096	8,462	11,620	17,058	23,543	45,124
Cumulative deaths, mean	S3 ^z^	763	3,743	5,276	7,303	11,537	16,538	121,960
Cumulative deaths, lower	S3 ^z^	434	2,095	3,067	4,203	6,537	9,895	42,697
Cumulative deaths, upper	S3 ^z^	1,254	6,096	8,462	11,620	17,058	23,543	252,429
Cumulative deaths, mean	S4 ^aa^	744	3,616	5,089	7,053	11,102	15,908	26,738
Cumulative deaths, lower	S4 ^aa^	388	1,777	2,572	3,590	5,960	9,019	16,176
Cumulative deaths, upper	S4 ^aa^	1,127	5,712	8,112	10,778	16,171	22,470	35,621
Cumulative deaths, mean	S5 ^bb^	744	3,616	5,089	7,053	11,102	15,908	31,916
Cumulative deaths, lower	S5 ^bb^	388	1,777	2,572	3,590	5,960	9,019	18,504
Cumulative deaths, upper	S5 ^bb^	1,127	5,712	8,112	10,778	16,171	22,470	48,300
Cumulative deaths, mean	S6 ^cc^	744	3,616	5,089	7,053	11,102	15,908	85,087
Cumulative deaths, lower	S6 ^cc^	388	1,777	2,572	3,590	5,960	9,019	40,819
Cumulative deaths, upper	S6 ^cc^	1,127	5,712	8,112	10,778	16,171	22,470	158,299
- LANL [14]								
Cumulative deaths, median	S1^i^	1,284	5,118	7,183	9,507	14,634	20,376	34,263
Cumulative deaths, lower	S1^i^	1,284	5,118	7,183	9,507	14,634	20,376	30,762
Cumulative deaths, upper	S1^i^	1,284	5,118	7,183	9,507	14,634	20,376	43,022
- Mashayekhi [28]								
Cumulative deaths	S1 ^dd^	759	10,316	11,751	11,857	··	··	··
Cumulative deaths	S2 ^ee^	1,285	33,349	61,322	77,302	86,931	92,620	··
Cumulative deaths	S3 ^ff^	11,752	97,445	612,953	1,819,392	3,002,721	3,562,136	··
- Moghadami [36]								
Cumulative deaths, mean^gg^	S1^i^	1,144	5,378	··	··	··	··	··
Cumulative deaths, lower^gg^	S1^i^	1,104	3,929	··	··	··	··	··
Cumulative deaths, upper^gg^	S1^i^	1,166	7,003	··	··	··	··	··
- Rafieenasab [54]								
Cumulative deaths	S2 ^hh^	32,101	39,026	··	··	··	··	··
Cumulative deaths	S3 ^ii^	69,583	388,951	402,569	··	··	··	··
- Srivastava [15]								
Cumulative deaths	S1P1 ^jj^	··	··	··	··	··	··	43,631
Cumulative deaths	S1P2 ^kk^	··	··	··	··	··	··	43,282
Cumulative deaths	S1P5 ^ll^	··	··	··	··	··	··	42,289
Cumulative deaths	S1P10 ^mm^	··	··	··	··	··	··	40,802
Cumulative deaths	S1P20 ^nn^	··	··	··	··	··	··	38,324
Cumulative deaths	S1P40 ^oo^	··	··	··	··	··	··	34,721
Cumulative deaths	S2P1 ^pp^	··	··	··	··	··	··	418,834
Cumulative deaths	S2P2 ^qq^	··	··	··	··	··	··	354,756
Cumulative deaths	S2P5 ^rr^	··	··	··	··	··	··	241,214
Cumulative deaths	S2P10 ^ss^	··	··	··	··	··	··	154,826
Cumulative deaths	S2P20 ^tt^	··	··	··	··	··	··	87,664
Cumulative deaths	S2P40 ^uu^	··	··	··	··	··	··	45,995
Cumulative deaths	S3P1 ^vv^	··	··	··	··	··	··	27,959
Cumulative deaths	S3P2 ^ww^	··	··	··	··	··	··	27,786
Cumulative deaths	S3P5 ^xx^	··	··	··	··	··	··	27,327
Cumulative deaths	S3P10 ^yy^	··	··	··	··	··	··	26,724
Cumulative deaths	S3P20 ^zz^	··	··	··	··	··	··	25,909
Cumulative deaths	S3P40 ^aaa^	··	··	··	··	··	··	25,043

^a^ Date 1: Gregorian

^b^ Date 2: Hijri

^c^ Latest date in 2020: As of 2020-10-19 for MOHME official via ([4, 5]), 2020-11-01 for Gu (YYG) [17], 2020–12–31 for IHME [12] and Imperial [13] [2020-11-28 for LANL [14], and 2020-12-19 for Srivastava [15]

^d^ S/M: Scenario / Model

^e^ N/A: Not Applicable

^f^ M1: Gompertz

^g^ M2: Von Bertalanffy growth

^h^ M3: Cubic Polynomial

^i^ S1: Single scenario

^j^ S1P1: Seasonality conditions 1 (no effect or status quo) and Policy effect 1 (status quo contact rate). Estimates for 2020-03-19, the end of first month after the epidemic start date, are equal across the six scenarios

^k^ S1P2: Seasonality conditions 1 (no effect or status quo) and Policy effect 2 (aggressive efforts to decrease contact rate by half of what it would be otherwise)

^l^ S2P1: Seasonality conditions 2 (moderate effect; infectivity of the virus decreases linearly from April 1st and halves by June 1st, then stays the same for the rest of the simulation) and Policy effect 1 (status quo contact rate)

^m^ S2P2: Seasonality conditions 2 (moderate effect; infectivity of the virus decreases linearly from April 1st and halves by June 1st, then stays the same for the rest of the simulation) and Policy effect 2 (aggressive efforts to decrease contact rate by half of what it would be otherwise)

^n^ S3P1: Seasonality conditions 3 (very strong mitigating effect; infectivity of the virus decreases from April 1st to a quarter of its base value by June 1st, then stays the same for the rest of the simulation) and Policy effect 1 (status quo contact rate)

^o^ S3P2: Seasonality conditions 3 (very strong mitigating effect; infectivity of the virus decreases from April 1st to a quarter of its base value by June 1st, then stays the same for the rest of the simulation) and Policy effect 2 (aggressive efforts to decrease contact rate by half of what it would be otherwise)

^p^ S0: Basic scenario (no intervention), only 10% isolation

^q^ S1: Worst scenario, minimum (25%) isolation

^r^ S2: Medium scenario, medium (32%) isolation

^s^ S3: Best scenario, maximum (40%) isolation

^t^ Smoothed estimates

^u^ S1 Best (Masks): ‘Universal Masks’ scenario reflects 95% mask usage in public in every location

^v^ S2 Reference (Current): ‘Current projection’ scenario assumes social distancing mandates are re-imposed for 6 weeks whenever daily deaths reach 8 per million (0.8 per 100,000)

^w^ S3 Worse (Easing): ‘Mandates easing’ scenario reflects continued easing of social distancing mandates, and mandates are not re-imposed

^x^ S1: Additional 50% Reduction

^y^ S2: Maintain Status Quo

^z^ S3: Relax Interventions 50%

^aa^ S4: Surged Additional 50% Reduction

^bb^ S5: Surged Maintain Status Quo

^cc^ S6: Surged Relax Interventions 50%

^dd^ S1: Serious distancing

^ee^ S2: Not serious distancing

^ff^ S3: Worse than Scenario 2

^gg^ Dates for Moghadami [36] are 2020-03-21 and 2020-04-18, instead of 2020-03-19 and 2020-04-19 respectively

^hh^ S2: Based on SIR model

^ii^ S3: Approximation calculation

^jj^ S1P1: Scenario Current, Parameter 1

^kk^ S1P2: Scenario Current, Parameter 2

^ll^ S1P5: Scenario Current, Parameter 5

^mm^ S1P10: Scenario Current, Parameter 10

^nn^ S1P20: Scenario Current, Parameter 20

^oo^ S1P40: Scenario Current, Parameter 40

^pp^ S2P1: Scenario Released, Parameter 1

^qq^ S2P2: Scenario Released, Parameter 2

^rr^ S2P5: Scenario Released, Parameter 5

^ss^ S2P10: Scenario Released, Parameter 10

^tt^ S2P20: Scenario Released, Parameter 20

^uu^ S2P40: Scenario Released, Parameter 40

^vv^ S3P1: Scenario Restricted, Parameter 1

^ww^ S3P2: Scenario Restricted, Parameter 2

^xx^ S3P5: Scenario Restricted, Parameter 5

^yy^ S3P10: Scenario Restricted, Parameter 10

^zz^ S3P20: Scenario Restricted, Parameter 20

^aaa^ S3P40: Scenario Restricted, Parameter 40

**Table 3:** Lowest and highest predictions at the end of month 2 (2020-04-19), month 4 (2020-06-20) after the official epidemic start date (2020-02-19), and the latest dates available in 2020 and 2021

Outcomes:	Lowest	Study	MOHME	Highest	Study
**- End of month 2 (20–04-19)**					
Cumulative deaths	1,777	Imperial ^a^	5,118	388,951	Rafieenasab ^b^
Daily deaths	30	Imperial ^a^	87	11,289	Rahimi Rise ^c^
Cumulative cases	20,588	Al-Qaness ^d^	82,211	2,310,161	IHME ^e^
Incident daily cases	93	Thu ^f^	1,343	216,262	Rahimi Rise ^c^
Incident daily total cases ^g k^	72,950	Saberi (paper) ^h^	..	1,616,385	Saberi (paper) ^i^
**- End of month 4 (20–06-20)**					
Cumulative deaths	3,590	Imperial ^a^	9,507	1,819,392	Mashayekhi ^j^
Daily deaths	5	Mashayekhi ^k^	115	44,934	Mashayekhi ^j^
Cumulative cases	144,305	DELPHI ^l^	202,584	4,266,964	IHME ^e^
Incident daily cases	211	DELPHI ^l^	2,322	138,892	Gu (YYG) ^m^
Incident daily total cases ^g^	9,625	Saberi (paper) ^h^	..	1,255,012	Saberi (paper) ^i^
**- Latest date available in 2020**					
Cumulative deaths	16,176	Imperial ^n^	30,712 ^o^	418,834	Srivastava ^p^
Daily deaths	0	Imperial ^a^	373 ^o^	3,984	Imperial ^q^
Cumulative cases	3,588,293	Imperial ^n^	534,631^o^	41,475,792	Imperial ^q^
Incident daily cases	0	Imperial ^a^	4,251^o^	486,745	Imperial ^e^
Incident daily total cases ^g^	9,625	Saberi (paper) ^h^	..	169,110	Saberi (paper) ^i^
**- Latest date available in 2021**					
Cumulative deaths	40,151	IHME ^r^	..	125,690	IHME ^s^
Daily deaths	55	IHME ^r^	..	1,093	IHME ^s^
Cumulative cases	19,799,934	IHME ^r^	..	34,417,912	IHME ^s^
Incident daily cases	14,818	IHME ^r^	..	236,781	IHME ^s^

^a^ Imperial, S4: Surged Additional 50% Reduction. Lower 95% uncertainty limit [13]

^b^ Rafieenasab, S3: Approximation calculation. Mean estimate [54]

^c^ Rahimi Rise, S2: No interventions. Mean estimate [29]

^d^ Al-Qaness, M1: Adaptive Neuro-Fuzzy Inference System (ANFIS) enhanced with Genetic Algorithm (GA). Mean estimate [51]

^e^ IHME, S2 Reference (Current): ‘Current projection’ scenario assumes social distancing mandates are re-imposed for 6 weeks whenever daily deaths reach 8 per million (0.8 per 100,000). Upper 95% uncertainty limit [12]

^f^ Thu, M1: Linear growth rate, eq. 1. Mean estimate [48]

^g^ Saberi (paper), Incident daily total cases (confirmed and suspected) [22]

^h^ Saberi (paper), S1: 20% more distancing. Mean estimate [22]

^i^ Saberi (paper), S3: 20% less distancing. Upper 95% uncertainty limit [22]

^j^ Mashayekhi, S3: Worse than Scenario 2 (S2: Not serious distancing). Mean estimate [28]

^k^ Mashayekhi, S1: S1: Serious distancing. Mean estimate [28]

^l^ DELPHI, S1: Single scenario. Mean estimate [10]

^m^ Gu (YYG) S1, Single scenario. Upper 95% uncertainty limit [17]

^n^ Imperial, S4: Surged Additional 50% Reduction. Lower 95% uncertainty limit. For 2020-12-31 [13]

^o^ MOHME official via ([4, 5]), as of 2020-10-19

^p^ Srivastava, S2P1: Scenario Released, Parameter 1. mean estimate For 2020-12-19 [15]

^q^ Imperial, S3: Relax Interventions 50%. Upper 95% uncertainty limit. For 2020-12-31 [13]

^r^ IHME, S1Best (Masks): ‘Universal Masks’ scenario reflects 95% mask usage in public in every location. Lower 95% uncertainty limit. For 2021-01-31 [12]

^s^ IHME, S3 Worse (Easing): ‘Mandates easing’ scenario reflects continued easing of social distancing mandates, and mandates are not re-imposed. Upper 95% uncertainty limit. For 2021-01-31 [12]

**Fig. 1:**
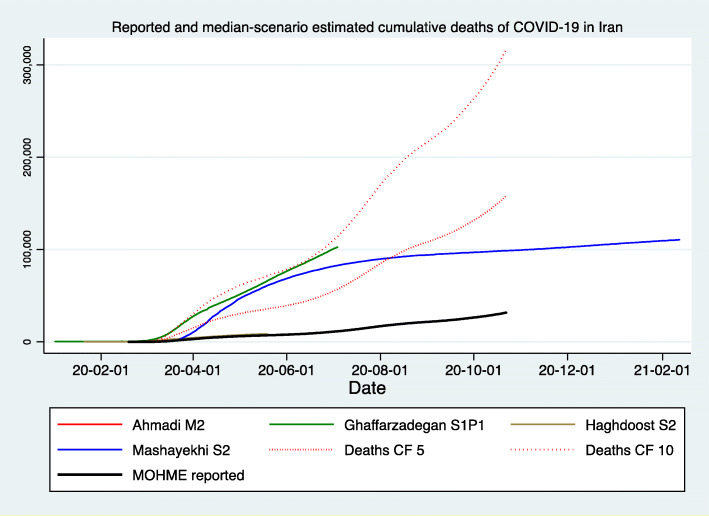
Reported and median-scenario estimated cumulative deaths of COVID-19 in Iran . (1) Ahmadi M2: Model 2, Von Bertalanffy (Curve lies behind MOHME reported) [44]. (2) Ghaffarzadegan S1P1: Seasonality conditions 1 (no effect or status quo) and Policy effect 2 (aggressive efforts to decrease contact rate by half of what it would be otherwise) [41]. (3) Haghdoost S2: Medium scenario, medium (32%) isolation (Curve lies behind MOHME reported) [27]. (4) Mashayekhi S2: Medium scenario, not serious distancing; People reduce their social [physical] contacts only to 20% of regular level, voluntarily, after number of cases and deaths have increased, and other settings are like scenario one [28]. (5) Deaths CF 5: Reported deaths with a Correction Factor of 5, after Dr. Rick Brennan, Director of Emergency Operations, World Health Organization [57]. (6) Deaths CF 10: Reported deaths with a Correction Factor of 10, after Russell [58]. (7) MOHME reported: Official reported deaths via [4, 5]

**Fig. 2:**
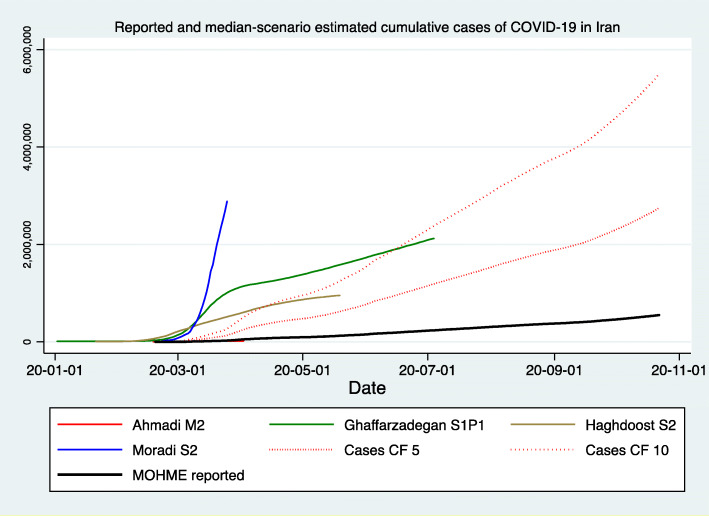
Reported and median-scenario estimated cumulative case of COVID-19 in Iran. (1) Ahmadi M2: Model 2, Von Bertalanffy (Curve lies behind MOHME reported) [44]. (2) Ghaffarzadegan S1P1: Seasonality conditions 1 (no effect or status quo) and Policy effect 2 (aggressive efforts to decrease contact rate by half of what it would be otherwise) [41]. (3) Haghdoost S2: Medium scenario, medium (32%) isolation [27]. (4) Moradi S2: Scenario 2, Case Fatality Rate, 0.5% [42]. (5) Cases CF 5: Reported cases with a Correction Factor of 5, after Dr. Rick Brennan, Director of Emergency Operations, World Health Organization [57]. (6) Cases CF 10: Reported cases with a Correction Factor of 10, after Russell [58]. (7) MOHME reported: Official reported cases via [4, 5]

**Fig. 3:**
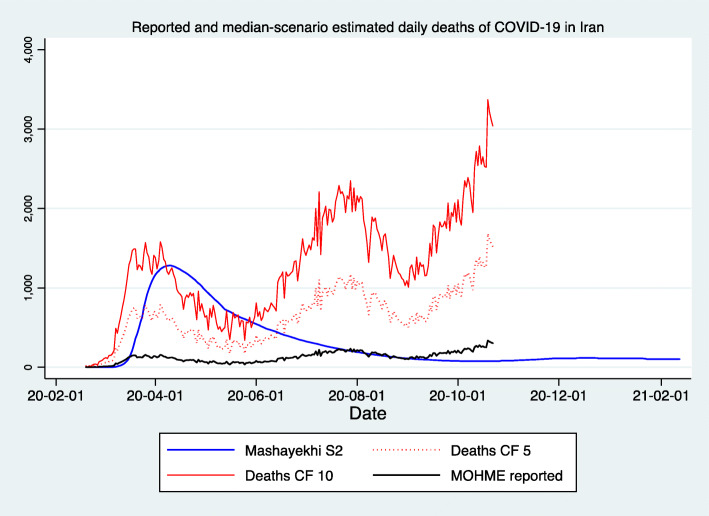
Reported and median-scenario estimated daily deaths of COVID-19 in Iran. (1) Mashayekhi S2: Medium scenario, not serious distancing; People reduce their social [physical] contacts only to 20% of regular level, voluntarily, after number of cases and deaths have increased, and other settings are like scenario 1 [28]. (2) Deaths CF 5: Reported deaths with a Correction Factor of 5, after Dr. Rick Brennan, Director of Emergency Operations, World Health Organization [57]. (3) Deaths CF 10: Reported deaths with a Correction Factor of 10, after Russell [58]. (4) MOHME reported: Official reported deaths via [4, 5]

**Fig. 4:**
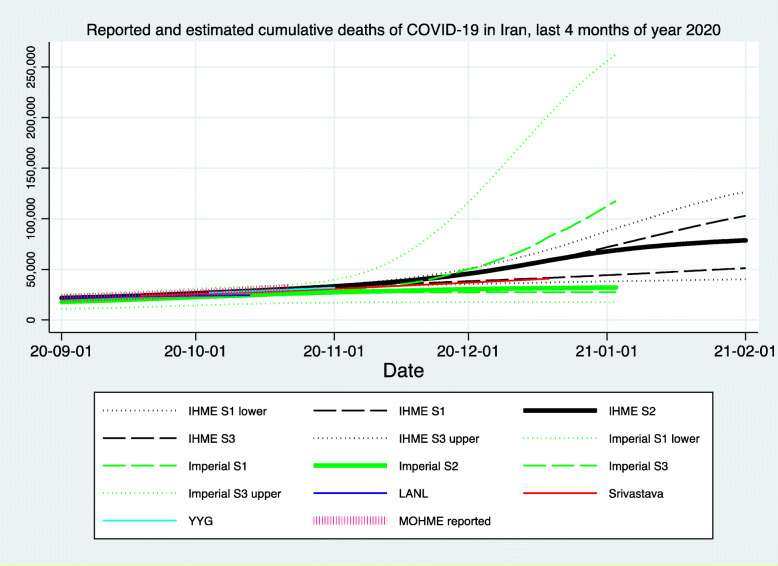
Reported and current (median) scenario cumulative deaths of COVID-19 in Iran, last 4 months of year 2020 and January 2021, International studies. (1) DELPHI: DELPHI (Differential Equations Leads to Predictions of Hospitalizations and Infections) Epidemiological Case Predictions. Mean estimate [10]. (2) IHME: Institute for Health Metrics and Evaluation (IHME) Mean estimate [12]. (3) Imperial: Imperial College COVID-19 LMIC Reports. Mean estimate [13]. (4) LANL: Los Alamos National Laboratory (LANL) COVID-19 Cases and Deaths Forecasts. Mean estimate [14]. (5) Srivastava: ReCOVER- Accurate Predictions and Resource Management for COVID-19 Epidemic Response. Mean estimate [15]. (6) YYG (Youyang Gu): COVID-19 Projections Using Machine Learning. Mean estimate [17]. (7) MOHME reported: Ministry of Health and Medical Education, Iran via [4, 5]

**Fig. 5:**
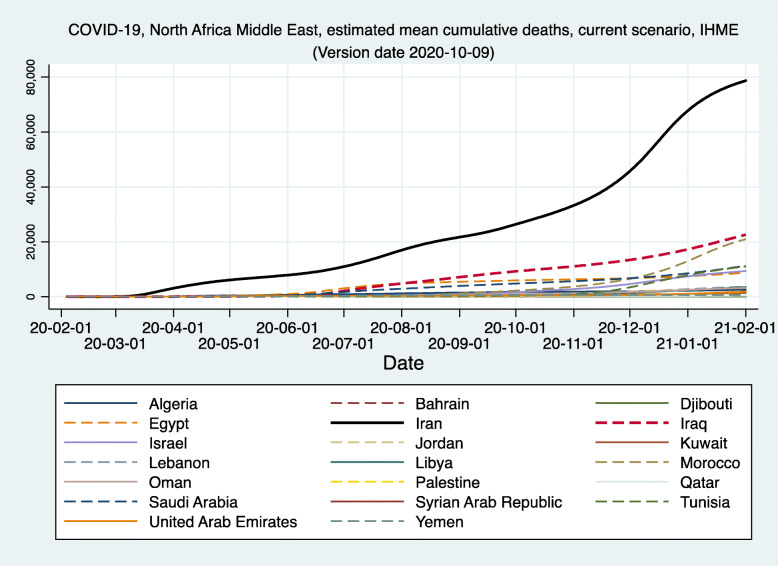
Current (median) scenario estimated cumulative deaths of COVID-19 in Iran and 20 other countries of North Africa Middle East, IHME. IHME: Institute for Health Metrics and Evaluation (IHME) Mean estimate. The Reference or ‘Current projection’ scenario assumes social distancing mandates are re-imposed for 6 weeks whenever daily deaths reach 8 per million (0.8 per 100,000) [12]

Figures 1, 2, and 3 demonstrate the reported and estimated outcomes in median scenarios. Figures 1 and 2 show the cumulative deaths and cumulative cases respectively. Figure 3 shows the daily deaths. In general, international studies tend to replicate or imitate the official national reports of deaths and cases up to the date that they are available and estimate the future trajectories for when the official reports seize to be available. Therefore, adding such estimates from the international studies does not provide added value. Hence, international studies’ estimates are not added to Figs. 1, 2 and 3. We created two graphs for the international studies’ estimates for Iran. The first one (Appendix Figure 8) shows officially reported and “current scenario” estimates of cumulative deaths for the whole time period available, starting on 2020-01-03 (Imperial [13]) and ending with 2021-02-01 (IHME [12]). The second graph for the international studies’ estimates for Iran is Fig. 4 and shows officially reported and “current scenario” estimates of cumulative deaths for the last 4 months of 2020 and January 2021, as well as highest and lowest estimates from other scenarios. Figure 5 shows the “current scenario” estimates of cumulative deaths by IHME for Iran and 20 other countries in North Africa Middle East region.

Appendix Figure 1 shows the PRISMA study flow diagram. Appendix Figure 2 demonstrates the officially reported cumulative confirmed cases, deaths, and recovered cases, and Appendix Figure 3 shows the daily equivalents. Appendix Figures 4 and 5 show the estimated daily prevalent cases, with and without the estimate form Saberi (web site) [21]. That estimate by Saberi, even in the median scenario, had high values compared to other studies. To visualize the quantitative diversity of the studies’ results, we also graphed the reported and worst-scenario estimated cumulative deaths in Appendix Figures 6 and 7, with and without the estimate form Mashayekhi [28]. That estimate by Mashayekhi, was the most extreme prediction among all the studies. Appendix Figure 8 shows officially reported and “current scenario” estimates of cumulative deaths by international studies for Iran.

#### MOHME

Official reports of MOHME for cumulative deaths and cases at 2020-10-19 were 30,712 and 534,631 respectively (via [4, 5]). Peaks in daily deaths (and dates) were 158 (2020-04-04), 235 (2020-07-28), and 337 (2020-10-19). Peaks in daily cases (and dates) were 3,186 (2020-03-30), 3,574 (2020-06-04), and 6,191 (2020-10-25).

#### Cumulative deaths

Lowest and highest predicted cumulative deaths for the end of the second month (2020-04-19) were 1777 (Imperial [13]) and 388,951 (Rafieenasab [54]) respectively, when the official number was 5,118. At the end of month four (2020-06-20), they were 3,590 (Imperial [13]) and 1,819,392 (Mashayekhi [28]), and the official number was 9,507. Those estimates for latest date available in 2020 were 16,176 (Imperial [13] for 2020-12-31) and 418,834 (Srivastava [15], for 2020-12-19). For the latest date available in 2021, those estimates were 40,151 (IHME [12], for 2021-01-31) and 125,690 (IHME [12], for 2021-01-31).

#### Cumulative cases

Lowest and highest predicted cumulative cases for the end of the second month (2020-04-19) were 20,588 (Al-Qaness [51]) and 2,310,161 (IHME [12]) respectively. Where the official number was 82,211. At the end of month four (2020-06-20), those estimates were 144,305 (DELPHI [10]) and 4,266,964 (IHME [12]), and the official number was 202,584. Those estimates for latest date available in 2020 were 3,588,293 (Imperial [13], for 2020-12-31) and 41,475,792 (Imperial [13], for 2020-12-31). For the latest date available in 2021, those estimates were 19,799,934 (IHME [12], for 2021-01-31) and 34,417,912 (IHME [12], for 2021-01-31).

#### Daily deaths

Lowest and highest predicted daily deaths for the end of the second month (2020-04-19) were 30 (Imperial [13]) and 11,289 (Rahimi Rise [29]) respectively, where the official number was 87. At the end of month four (2020-06-20), they were 5 (Mashayekhi [28]) and 44,934 (Mashayekhi [28]), and the official number was 115. Those estimates for latest date available in 2020 were zero (Imperial [13], for 2020-12-31) and 3,984 (Imperial [13], for 2020-12-31). For the latest date available in 2021, those estimates were 55 (IHME [12], for 2021-01-31) and 1,093 (IHME [12], for 2021-01-31).

#### Daily cases

Lowest and highest predicted daily incident cases for the end of the second month (2020-04-19) were 93 (Thu [48]) and 216,262 (Rahimi Rise [29]) respectively, where the official number was 1,343. At the end of month four (2020-06-20), they were 211 (DELPHI [10]) and 138,892 (Gu (YYG) [11]), and the official number was 2,322. Those estimates for latest date available in 2020 were zero (Imperial [13], for 2020-12-31) and 486,745 (Imperial [13], for 2020-12-31). For the latest date available in 2021, those estimates were 14,818 (IHME [12], for 2021-01-31) and 236,781 (IHME [12], for 2021-01-31). Lowest and highest predicted daily incident confirmed and suspected cases for the end of the second month (2020-04-19) were 72,950 (Saberi (article) [22]) and 1,616,385 (Saberi (article) [22]) respectively; there are no correspondent official numbers for this measure. At the end of month four (2020-06-20), they were 9,625 (Saberi (article) [22]) and 1,255,012 (Saberi (article) [22]).

#### Peak dates and control dates

In general, studies with shorter (or longer) durations of outputs estimated lower (or higher) numbers of peaks. For daily deaths, official reports showed the first peak of 158 (2020-04-04), second peak of 235 (2020-07-28), and third peak of 337 (2020-10-19). For estimates of daily deaths, size (and date) of the lowest first peak was 81 (2020-06-01) (LANL [14]), and the highest first peak was 44,934 (2020-06-20) (Mashayekhi [28]). For the third peak, lowest estimate was 134 (2020-09-29) (Imperial [13]) and highest estimate was 4,968 (2020-12-11) (Imperial [13]). For daily cases, official reports showed the first peak of 3,186 (2020-03-30), second peak of 3,574 (2020-06-04), and third peak of 4,830 (2020-10-14). For estimates of daily cases, size (and date) of the lowest first peak was 1,050 (2020-03-12) (Shen [43]), and the highest first peak was 470,229 (2020-03-31) (Rahimi Rise [29]). For the second peak, lowest estimate was 3,825 (2020-10-010) (LANL [14]) and highest estimate was 14,272 (2020-03-11) (Haghdoost [27]). For the third peak, lowest estimate was 6,526 (2020-10-25) (LANL [14]) and highest estimate was 717,356 (2020-12-04) (Imperial [13]).

Three studies predicted the epidemic control (or end) dates and outcome’s values. Two studies predicted the potential date for epidemic to be controlled in April; Ahmadi et al. predicted the “end of the epidemic” on 2020-05-13 with 87,000 cumulative cases or on 2020-06-01 with 4,900 cumulative deaths (using Von Bertalanffy model) or 11,000 cumulative deaths (using Gompertz model) [44]. Haghdoost predicted that with their either medium or best scenarios, the epidemic would be well controlled in month 2 of Hijri solar year 1,399 (2020-04-20 to 2020-05-20). Their ‘maximum number of infected people in day’ would be 92,100 in middle scenario and 9150 in best scenario [27]. Zhan et al. predicted that if the “authorities continue to impose strict control measures, the epidemic will come under control by the end of April and is expected to end before June 2020, and as the quality of treatment improves, more rapid recovery will be expected” [40]. Beyond the correspondent values of the predicted outcomes, no further criteria or definition of epidemic end or control was provided.

## Discussion

There were lots of heterogeneity in methods and findings of the COVID-19 prediction models and estimation studies for Iran. After the presumed official start date of the COVID-19 epidemic in Iran, i.e. 2020-02-19, and at the end of month two (2020-04-19), the lowest (and highest) values of predictions were 1777 (388,951) for cumulative deaths, 20,588 (2,310,161) for cumulative cases, and at the end of month four (2020-06-20), they were 3,590 (1,819,392) for cumulative deaths, and 144,305 (4,266,964) for cumulative cases. Lowest and highest predicted cumulative deaths for latest date available in 2020 were 16,176 (Imperial [13], for 2020-12-31) and 418,834 (Srivastava [15], for 2020-12-19). For the latest date available in 2021, those estimates were 40,151 (IHME [12], for 2021-01-31) and 125,690 (IHME [12], for 2021-01-31). Lowest and highest predicted cumulative cases for latest date available in 2020 were 3,588,293 (Imperial [13], for 2020-12-31) and 41,475,792 (Imperial [13], for 2020-12-31). For the latest date available in 2021, those estimates were 19,799,934 (IHME [12], for 2021-01-31) and 34,417,912 (IHME [12], for 2021-01-31).

Part of the heterogeneity observed in component studies’ methods originates from the actual lack of universal consensus-based standards for epidemic modeling study methodology and reporting. Another part originates from studies’ adherence to principles of reporting their methods and findings. For instance, eight of the reviewed studies did not mention their study limitations at all, and nine studies did mention limitations but just touched the limitations very minimally.

Part of the heterogeneity observed in studies’ findings originates from choices of methods and assumptions, including the untrue assumptions of homogeneous population mixing, no role of asymptomatic cases in disease spread, and no under-reporting of deaths and cases. Another part originates from quality, availability, and completeness of data, including, epidemic start date, and numbers of cases and deaths.

The epidemic start date and the reported number of deaths and cases are the most important starting points for epidemic estimation studies. There are uncertainties about the epidemic start date and real numbers of deaths and cases. The presumed official start date of the COVID-19 epidemic in Iran was 2020-02-19, when the first two tandem cases were reported as dead. Report of the first case or cases as dead on the same date they were diagnosed is not the most frequent type of reporting in this pandemic. Haghdoost et al. study, dated 2020-03-15 [1,398-12-25 Hijri solar], maintains that their start date of the epidemic in Iran for their modeling purpose was designated as 2020-01-21 [1,398-11-01 Hijri solar] based on “available documentations and epidemiologic analyses” [27]. No description or references were provided for their “available documentations and epidemiologic analyses”. Two days later, MOHME announced in 2020-03-27 that the epidemic had probably started in month 11 of Hijri solar year 1,398 (2020-01-21 to 2020-02-19) [59]. As such, the models that use the official start date of 2020-02-19 start with an inaccurate start date of the epidemic to begin with.

WHO Country Support Mission to Iran (2–11 March 2020) reported the following: “On 20 February, the Islamic Republic of Iran IHR [International Health Regulations] National Focal Point (IHR-NFP) notified WHO of five cases, including two deaths, of laboratory-confirmed COVID-19 cases. Three of the cases were from Qom City, and the fourth had a travel history to Qom. In the following days, the investigation concluded that the virus was probably circulating in Qom for several weeks, based on the following observations: Among 186 patients with severe acute severe acute respiratory infection (SARI) hospitalized during February, 8 deaths were observed (0 deaths for the same month last year). Samples taken in February in patients with influenza-like illness (ILi) symptoms that tested negative for Influenza were also tested for COVID-19. Among workers of the Salafchegan free zone located 50 km from Qom city centre, 5 tests were positive for COVID-19; their onset of symptoms was 10 February. In late February, of 17 Chinese workers who had not traveled back to China for the Chinese New Year, 5 tested positive.” [57]. As such, most of the models start with an inaccurate start date of the epidemic to begin with, and most of the studies rely on the officially reported numbers of cases and deaths. Iran’s MOHME stopped reporting provincial cases and deaths on 2020-03-23 [45].

In addition to Ghaffarzadegan [41] that used both official and unofficial data as input, one study (Saberi [21]), also used correction factors of 5 and 10 taken from other sources [57] or studies [58] applied to the officially reported numbers of cases and deaths. A correction factor of 20 has been proposed for the epidemic in Iran [60]. We do not know when or where were the results of the ‘investigation’ referred to in the above quoted “In the following days, the investigation concluded that …” , were announced or published. The first COVID-19 patient “with a definite diagnosis” was reported in an article by Ghadir and colleagues on 2020-04-06 [61]. On 2020-07-30, clinical and virologic characteristics of the first seven cases of COVID-19 in Iran were reported by Yavarian and colleagues, academics from Tehran University of Medical Sciences and officials from Iran MOHME [62]. Numbers and dates of cases and deaths do not match with correspondent numbers and dates of cases and deaths officially announced by MOHME. Date of symptoms onset and date of encounter for the first patient reported by Ghadir [61] and by Yavarian [62] do not match.

A study on all-cause excess mortality and COVID-19-related deaths in Iran found a correction factor of two for reported COVID-19 deaths in Iran [63]. Deputy Minister of Health in Iran announced on 2020-10-14 that the real numbers of COVID-19 deaths In Iran are on average about two times higher than the official reports (ranging 1.7 from 2.2 depending on the province), because they follow the WHO protocols that requires positive PCR test result [64]. World Health Organization maintains that’ “Countries have varying approaches to COVID-19 case definitions. Consequently, the numerator and the denominator of any formula used to calculate fatality rate will vary according to how they are defined. WHO recommends using the surveillance case definitions which are available in the WHO interim guidance on Global surveillance for COVID-19. A COVID-19 death is defined for surveillance purposes as a death resulting from a clinically compatible illness in a probable or confirmed COVID-19 case, unless there is a clear alternative cause of death that cannot be related to COVID-19 disease (e.g. trauma). There should be no period of complete recovery between the illness and death.” [65]. Moreover, WHO’s “International Guidelines for Certification and Classification (Coding) of Covid-19 as Cause of Death” maintains the immediately-above-mentioned for a “COVID-19 death”, and also provide the following codes. “U07.1 COVID-19, virus identified” for when the virus was identified, and “U07.2 COVID-19, virus not identified. Clinically-epidemiologically diagnosed COVID-19. Probable COVID-19. Suspected COVID-19” for when the virus was NOT identified [66]. However, WHO maintains in its “COVID-19 Weekly Epidemiological Update” of 25 October 2020, that “Data presented are based on official laboratory-confirmed COVID-19 case and deaths reported to WHO by country/territories/areas, *largely* based upon WHO case definitions and surveillance guidance.” and “A *small* number of countries/territories/areas report combined probable and laboratory-confirmed cases; efforts are underway to identify these for notation in the data table. Differences are to be expected between information products published by WHO, national public health authorities, and other sources.” [67].

Another deputy Minister of Health in Iran announced on 2020-10-18 that the real numbers of COVID-19 deaths and cases of COVID-19 In Iran are on average about 2.5 times more than the official reports because the diagnostic test kits have only 30 to 50% ability to diagnose the disease [68]. A member of High Council of Iranian Medial Council mentioned on 2020-10-25 that the real number of COVID-19 deaths is 3–4 times more than the official reports due to low number of PCR testing [69]. Euro-news quoted Dr. Michael Ryan of WHO on 2020-10-05, “Around 10% of the world’s population may have had COVID-19” on about 2020-10-05 [70]. There were 37,738,990 reported global cumulative cases on 2020-10-05 [49], and 10% of 7.8 billion global population is 780 million, and hence the correction factor for going from reported cases to the total cases is 20.67 at the global level. If the global average Infection Fatality Rate (IFR) is about 0.5% (0.2–1.0%) [71–74], then with 780 million expected global cumulative deaths on 2020-10-05, the expected deaths could be 3,900,000 (1,560,000-7,800,000). With 1,054,089 reported global cumulative deaths on 2020-10-05, the correction factor for deaths would be about 3.70 (1.48–7.40) at the global level. Therefore, at the global average level, the correction factor for cases is about 20.67, and correction factor for deaths is about 3.70 (1.48–7.40). Deputy Minister of Health in Iran announced on 2020-10-25 [75] that based on a national seroprevalence study, 30 million people were infected at about the end of Hijri month 2 (about 2020-05-20). If the global average Infection Fatality Rate (IFR) is about 0.5% (0.2–1.0%) [71–74], and applies to Iran, then 30 million infections translates to 150,000 (60,000-300,000) deaths. Official report of cumulative deaths on 2020-05-20 was 7183, and all of these end up in three possibilities: (1) correction factor of 21 (8–42) for COVID-19 deaths in Iran, or (2) the IFR in Iran was 0.024% at that time, or (3) that seroprevalence finding was wrong. While some politicians and researchers may wish, advocate, or publish for herd immunity – knowingly or unknowingly [76–79], research evidence yet describe why “COVID-19 herd immunity is unethical and unachievable” [80, 81]. On a rather daily basis, more and more announcements are being made by authorities and officials about higher correction factors for COVID-19 deaths and cases in Iran, or unpreparedness of testing system or hospital infrastructure for combating the epidemic; for which continued quotation and referencing here would not further help anyone.

Undercounting, under-ascertainment, or under-reporting is a known issue with the number of official confirmed cases and deaths, almost in all countries. Factors such as health system capacity for performing tests, access of people to testing services, on-time availability of test results, precision of diagnostic or screening tests, performance of surveillance systems, and transparency of health systems affect number of cases and deaths in official reports. In addition to such factors, SARS-CoV-2 itself has characteristics that might aggravate undercounting. A study on Santa Clara county in the United States revealed that prevalence, based on testing antibodies to SARS-CoV-2, is 50–85-fold more than the confirmed cases [82]. This pattern is different from other viruses of the Coronaviridae family, such as Middle East Respiratory Syndrome (MERS-CoV), with an estimated 25–50% asymptomatic to mild cases [83]. Some of the reviewed studies have estimated number of infected cases without excluding or mentioning asymptomatic cases. An implicit conclusion is that their numbers mainly refer to symptomatic cases, similar to the case mix of their input data. Different approaches have been used in studies to the issue of undercounting, under-ascertainment, or under-reporting. (1) Some studies have accounted for undercounting in their models: DELPHI [10], Ghaffarzadegan [41], Gu (YYG) [17], Saberi (web site) [21], Saberi (article) [22], and Srivastava [15]. (2) Some studies have not used number of confirmed deaths and / or cases as input data; Haghdoost [27], Mashayekhi [28], Rahimi Rise [29], Tuite [46], and Zhuang [47]. (3) In order to conclude that “In emerging epidemics, CFR indicator must not be used as a basis to judge the performance of a health system unless that epidemic condition has been clarified”, Moradi estimated the “actual number of COVID-19 cases in Iran based on different proposed scenarios for Case Fatality Rate [42]. (4) Other studies did nothing about the issue of undercounting, with or without mentioning it. To our best understanding, the most important issue that can drive the prediction models’ results misleading and misinforming is ignoring the issue of undercounting, under-ascertainment, and under-reporting. Role of the symptomatic cases in spread of the disease was accounted for only in some studies; DELPHI [10], Ghaffarzadegan [41], Gu (YYG) [17], Mashayekhi [28], Saberi (article) [22], Rahimi Rise [29], Srivastava [15].

Some studies provided some sort of subnational estimates as well as national; Haghdoost [27], Moghadami [36], Muniz-Rodriguez [37], Pourghasemi (PLoS ONE) [38], Pourghasemi (IJID) [39], and Zhan [40]. Access to COVID-19 data at provincial and subnational level has obviously been an important limitation for most researchers. This threatens the usability of models. Naturally, all the provinces are not at the same stage of epidemic growth, they have different conditions that affect disease transmission and their capacities to respond to the epidemic are different. This means that centralized strategies for estimation of the epidemic extent and intervention options might not fit all the needs of the subnational levels. Spatial heterogeneity in propagation of epidemics should be taken into account [84]. MOHME has a subnational level defined between the national and the provincial levels; some of the studies or operational plans have used these conglomerates of provinces in Iran, labeled as “climes”, which share relatively homogenous epidemiologic profiles within the climes before the COVID-19 era. Such conglomerates of provinces in Iran, or newly designed conglomerates, might be considered usable in estimation of the epidemic propagation in Iran with a smaller number of subnational geographic units (i.e. climes), compared to studying all the provinces, which is more resource-intensive. Alternatively, there are potential modeling approaches for simultaneous modeling of different subnational levels accounting for different stages of epidemic progression, both with and without access to detailed province data; for instance, Rojas [85] and Xiong [86].

The international studies, i.e. DELPHI [10], Gu (YYG) [17], IHME [12], Imperial [13], LANL [14], and Srivastava [15], generally provide updated estimates on a periodical basis, mostly weekly. This means that all estimations (from the start date of the epidemic) change in each version of running model. The usual explanation for this approach is to feed model with more input data that can improve prediction and provide an opportunity for improving methods as well.

Among the models that have used number of confirmed cases or deaths as input, only two studies, Ghaffarzadegan [41] and Saberi (article) [22], have considered delayed diagnosis in their calculations; this might be quite important. The Wuhan municipal headquarters for COVID-19 epidemic prevention and control released a notification and revised the total number of fatalities up by around 50% to 3869 after reviewing all available sources of data [87]. There not an unusual practice in many of death registries that physicians use more general terms as the final diagnosis or cause of death when they cannot or do not have time to match patients’ characteristics with exact definitions. It is more common in situations like epidemics that all health care workers are overwhelmed with number of patients and preoccupied with treating patients. Also, field hospitals and COVID-19 specific hospices might not be linked properly to health information systems to share data. Some cases that were initially classified under more general terms (such as pneumonia or acute respiratory syndrome) or even more specified but incorrect diagnoses (such as seasonal flu) might be re-classified to COVID-19 after reviewing all clinical data, test results, or autopsies. Five studies (Ayyoubzadeh [52], Ghaffarzadegan [41], Haghdoost [27], Mashayekhi [28], Rahimi Rise [29]) forecasted more than one peak for the epidemic. International studies generally ‘back-cast’ (replicate or imitate) the officially reported epidemic curves up to the time where reported data is available, and ‘forecast’ the epidemic curve under different scenarios for the future. Prediction of time and magnitude of future waves of the epidemic is an important aspects of modeling studies. Also, there is an implicit assumption in all studies or models, that the socio-economic response capacity will remain constant over the timespan of the epidemic and the calendar time period for which estimations are performed; this is not necessarily correct.

As some of the studies have not mentioned enough details on their epidemic model and statistical methods, the largest gap was related to not mentioning the methods used to assess model validity, accuracy, or fitness, and the findings of these methods. Eleven studies did not report any method used to verify model validity. Absence of reporting uncertainty intervals for model output was another downside in statistical methods. Poor reporting has been one of the common issues in many of the COVID-19 prediction and estimation studies [7]. Although guidelines such as TRIPOD are not specifically designed for this type of studies but can be used as a base to remind researchers about the standards of reporting [9]. There have been a few systematic reviews on COVID-19 epidemiological studies. None of the studies included in this research were among the included studies in Park’s systematic review [88].

Eight studies (DELPHI [10], Ghaffarzadegan [41], Haghdoost [27], Hsiang [45], Imperial [13], Mashayekhi [28], Rahimi Rise [29], Thu [48]) have considered scenarios to assess the effects of social distancing policies; this increases the usability of these models, however social distancing policies have a wide range of methods and effectiveness. They need to be clarified with more details in scenarios to increase practical usability of models for decision making. We expect to see postponement in the first peak date or reduction of its height (i.e. flattening the curve) in scenarios based on appropriate interventions [35]. Most of the studies have ignored availability and numbers of performed tests to detect COVID-19 cases; such data were not publicly available in the first few weeks after start of the epidemic in Iran. Only two studies (Ghaffarzadegan [41], IHME [12]) included test coverage in their model. Evidence on potential seasonality effects is not conclusive yet, but it has been proposed by some researchers [36, 37]. This factor has been considered in three of the models; Ghaffarzadegan [41], Haghdoost [27], and IHME [12]. Among the 84 study-model/scenarios, the median scenario of Haghdoost [27] was the best model that perfectly forecasted the cumulative deaths officially reported by MOHME (Fig. 1).

Non-COVID causes of mortality and morbidity are also important in epidemic modeling and intervention planning. Increase in non-COVID all-cause mortality and morbidity is a tandem phenomenon running alongside the COVID epidemic, that goes on with less drawn attention compared to the epidemic. European mortality monitoring (EuroMOMO) Network has assessed excess all-cause mortality overall for the participating European countries and estimated a marked increase [38]. As the cold season will come, taking into account the influenza season in estimations, and in particular regarding the caseload burden to be imposed on the health care system. Some COVID cases or deaths might be misclassified as non-COVID Acute Respiratory Distress Syndrome (ARDS), influenza, or pneumonia, and analyses of expected levels of such cases deaths could illuminate and improve COVID estimates. Some countries have expedited release of reports of provisional counts of death and excess deaths in January to March 2019 and January to March 2020, e.g. Iran [39]. Excess deaths in provinces of Iran have been assessed in non-peer-reviewed report [40].

Models differ in their mathematical configuration, designated start date of the epidemic for a given population, use of parameter values as input (e.g. Basic reproduction number, R0; or Case Fatality Ratio, CFR), use of data with varying time lengths to calibrate the model, and interventions formulated in scenarios. As Panovska-Griffiths explains, the ultimate questions of “can mathematical modelling solve the current Covid-19 crisis” or “which model is correct” evolve to realization that “no one model can give all the answers” and that “we need more models that answer complementary subquestions that can piece together the jigsaw and halt COVID- 19 spread” [41]. Estimation and use of a single correction factor for both the cases and deaths all across the time for any given country assumes invariance of diagnosis, detection and reporting completeness for infections and mortality during the epidemic; which is not necessarily true.

We believe that increasing public access to data on number of confirmed or suspected cases (and their outcomes of death and recovery), healthcare utilization by patients with COVID-19 (such as hospital admission, intensive care utilization and performed tests) both at national and province levels may improve accuracy and usability of models, and eventually lead to prevention of more cases and deaths. Estimates of cumulative deaths are less dependent on testing compared to cumulative cases. Estimates of daily deaths and daily cases look at possible waves and peak heights. Estimation of all these four outcomes can depict a better trajectory and extent of the epidemic. We suggest researcher to consider subnational estimations, as well as factors such as non-pharmacological interventions, test availability, age-stratification, and delayed diagnosis for updating their models. Some of the reports do not have enough details on methods and results that reduces their usage in disease control. We recommend researchers to consider standard items for reporting their models to increase practical use of the findings. It would also be vitally desirable if the veteran and current researchers of epidemic modeling could develop a consensus-based declaration on Preferred Reporting Items of Epidemic Modeling Studies, preferably before the next pandemic.

The results of epidemic modeling studies are certainly uncertain. The way in which the problem of uncertainty is handled by some of the national-level and international models of COVID-19 epidemic has been criticized in Davey Smith’s ‘Covid-19’s known unknowns’ [89], albeit without suggestion of any solutions beyond “respecting uncertainty”. Beyond the considerations of usability and certainty of epidemic models, the immense problem for people on the ground who suffer from the direct and indirect mortality, morbidity, and economic hardship from COVID-19, is the actually materialized accountability and the public health and economic interventions the governments have undertaken, are doing, and will perform – with or without the best or worst estimation models’ results. As demonstrated in Fig. 5, Iran would have the highest estimated death toll among the 21 countries in in North Africa Middle East region, with 78,693 estimated cumulative deaths under the current scenario on 2021-02-01, that is about 3.5 times more than the second highest death toll of 22,614 in Iraq [12]. Model estimates predict an ominous course of epidemic progress in Iran. Wisdom, political and economic commitment, and effective policy making, and management are needed indeed.

### Study limitations

We did not search international databases other than PubMed, did not describe subnational results, and did not assess other outcomes such as utilization of intensive care unit (ICU) beds. We did not have access to the full report of one of the studies (Mashayekhi [28]). In the abridged study report we found, we noticed some discrepancies in outcome prediction values in different graphs [26]. None of our digitized outcome predictions reported here are 100% accurate. All of them are wrong in terms of having non-prefect accuracy. However, the error range is mostly around 1 or 2% points and below 5% for almost all instances.

## Conclusions

We believe that COVID-19 models which consider scenarios for policy options, include key influencing factors such as role of asymptomatic cases in spread of the disease, under-reporting of deaths and cases, testing availability, and delayed diagnosis, and provide estimates for subnational regions are more useful for epidemic control. Not accounting for under-reporting drives the models’ results misleading. Increasing public access to COVID-19 related data is very important for improving quality of models and enhancing evidence-informed decisions to prevent more deaths. To increase the usability of reports, researchers should consider requirements of reporting a prediction or estimation model.

## Supplementary Information

**Additional file 1: Appendix Text 1.** Search syntax used in PubMed. **Appendix Text 2.** Details of studies’ scenario. **Appendix Table 1.** Predictions of cumulative cases for the end of months one to six after the official epidemic start date (2020-02-19) and the latest date available in 2020. **Appendix Table 2.** Predictions of daily deaths at end of months one to six after the official epidemic start date (2020-02-19) and the latest date available in 2020. **Appendix Table 3.** Predictions of daily cases for the end of months one to six after the official epidemic start date (2020-02-19) and the latest date available in 2020. **Appendix Table 4.** Predictions of epidemic peak dates and values of outcomes. **Appendix Table 5.** Predictions of epidemic control dates and values of outcomes. **Appendix Figure 1.** PRISMA 2009 study flow diagram. **Appendix Figure 2.** Officially reported cumulative confirmed cases, deaths, and recovered cases of COVID-19 in Iran. **Appendix Figure 3.** Reported daily confirmed cases, deaths, and recovered cases of COVID-19 in Iran. **Appendix Figure 4.** Reported and median-scenario estimated daily prevalent cases of COVID-19 in Iran, including predictions by Saberi. **Appendix Figure 5.** Reported and median-scenario estimated daily prevalent case of COVID-19 in Iran, without predictions by Saberi. **Appendix Figure 6.** Reported and worst-scenario estimated cumulative deaths of COVID-19 in Iran, including predictions by Mashayekhi. **Appendix Figure 7.** Reported and worst-scenario estimated cumulative deaths of COVID-19 in Iran, without predictions by Mashayekhi. **Appendix Figure 8.** Reported and current (median) scenario estimated cumulative deaths of COVID-19 in Iran, International studies.

**Additional file 2.** Target studies’ abstracted data.

## Data Availability

All data generated or analysed during this study are included in this published article and its supplementary information files.

## Abbreviations

CCPV: Characteristics, Construction, Parameterization and Validation
CFR: Case Fatality Ratio
COVID-19: Coronavirus Disease 2019
ICU: Intensive Care Unit
DELPHI: Differential Equations Leads to Predictions of Hospitalizations and Infections (DELPHI) Epidemiological Case Predictions
Gu (YYG): (First author, Youyang Gu) COVID-19 Projections Using Machine Learning
IHME: Institute for Health Metrics and Evaluation. COVID-19 projections
IHR-NFP: International Health Regulations - National Focal Point
Ili: Influenza-like illness
Imperial: Imperial College COVID-19 LMIC Reports
LANL: Los Alamos National Laboratory (LANL) COVID-19 Cases and Deaths Forecasts
MERS-CoV: Middle East Respiratory Syndrome – Corona Virus
MOHME: Ministry of Health and Medical Education (of Iran)
PCR: Polymerase Chain Reaction (test)
PRISMA: Preferred Reporting Items for Systematic Reviews and Meta-Analyses
R0: Basic reproduction number
SARI: Acute Severe acute Respiratory Infection
SARS-CoV-2: Severe acute respiratory syndrome coronavirus 2
TRIPOD: Transparent Reporting of a multivariable prediction model for Individual Prognosis Or Diagnosis
WHO: World Health Organization
