# Supplementary material for: Rapid review of COVID-19 epidemic estimation studies for Iran
Source: BMC Public Health. 2021 Feb 1;21:257. doi: 10.1186/s12889-021-10183-3 (PMC7848865; doi:10.1186/s12889-021-10183-3)
Supplement: Supplementary file 1 — Additional file 1: Appendix Text 1. Search syntax used in PubMed. Appendix Text 2. Details of studies’ scenario. Appendix Table 1. Predictions of cumulative cases for the end of months one to six after the official epidemic start date (2020-02-19) and the latest date available in 2020. Appendix Table 2. Predictions of daily deaths at end of months one to six after the official epidemic start date (2020-02-19) and the latest date available in 2020. Appendix Table 3. Predictions of daily cases for the end of months one to six after the official epidemic start date (2020-02-19) and the latest date available in 2020. Appendix Table 4. Predictions of epidemic peak dates and values of outcomes. Appendix Table 5. Predictions of epidemic control dates and values of outcomes. Appendix Figure 1. PRISMA 2009 study flow diagram. Appendix Figure 2. Officially reported cumulative confirmed cases, deaths, and recovered cases of COVID-19 in Iran. Appendix Figure 3. Reported daily confirmed cases, deaths, and recovered cases of COVID-19 in Iran. Appendix Figure 4. Reported and median-scenario estimated daily prevalent cases of COVID-19 in Iran, including predictions by Saberi. Appendix Figure 5. Reported and median-scenario estimated daily prevalent case of COVID-19 in Iran, without predictions by Saberi. Appendix Figure 6. Reported and worst-scenario estimated cumulative deaths of COVID-19 in Iran, including predictions by Mashayekhi. Appendix Figure 7. Reported and worst-scenario estimated cumulative deaths of COVID-19 in Iran, without predictions by Mashayekhi. Appendix Figure 8. Reported and current (median) scenario estimated cumulative deaths of COVID-19 in Iran, International studies. [file 12889_2021_10183_MOESM1_ESM.docx]

**Additional files**

**File name: Additional file 1 - Appendix.docx**

**Title of data**: Appendix

**Description of data**: Contains the following.

Appendix Text 1. Search syntax used in PubMed

Appendix Text 2. Details of studies’ scenarios

Appendix Table 1. Predictions of cumulative cases for the end of months one to six after the official epidemic start date (2020-02-19) and the latest date available in 2020

Appendix Table 2. Predictions of daily deaths at end of months one to six after the official epidemic start date (2020-02-19) and the latest date available in 2020

Appendix Table 3. Predictions of daily cases for the end of months one to six after the official epidemic start date (2020-02-19) and the latest date available in 2020

Appendix Table 4. Predictions of epidemic peak dates and values of outcomes

Appendix Table 5. Predictions of epidemic control dates and values of outcomes

Appendix Figure 1. PRISMA 2009 study flow diagram

Appendix Figure 2. Officially reported cumulative confirmed cases, deaths, and recovered cases of COVID-19 in Iran

Appendix Figure 3. Reported daily confirmed cases, deaths, and recovered cases of COVID-19 in Iran

Appendix Figure 4. Reported and median-scenario estimated daily prevalent cases of COVID-19 in Iran, including predictions by Saberi

Appendix Figure 5. Reported and median-scenario estimated daily prevalent case of COVID-19 in Iran, without predictions by Saberi

Appendix Figure 6. Reported and worst-scenario estimated cumulative deaths of COVID-19 in Iran, including predictions by Mashayekhi

Appendix Figure 7. Reported and worst-scenario estimated cumulative deaths of COVID-19 in Iran, without predictions by Mashayekhi

Appendix Figure 8. Reported and current (median) scenario estimated cumulative deaths of COVID-19 in Iran, International studies

**Appendix Text 1. Search syntax used in PubMed**

((Iran[Title/Abstract])) AND (((COVID[Title/Abstract]) OR (COVID-19[Title/Abstract]) OR (COVID 19[Title/Abstract]) OR (Corona[Title/Abstract]) OR (SARS-CoV-2[Title/Abstract])) AND ((Epidemic[Title/Abstract]) OR (Outbreak[Title/Abstract]) OR (Pandemic[Title/Abstract]) OR (case*[Title/Abstract]) OR (death*[Title/Abstract]) OR (fatal*[Title/Abstract]) OR (mortalit*[Title/Abstract])) AND ((Model*[Title/Abstract]) OR (estimat*[Title/Abstract]) OR (predict*[Title/Abstract]))).

**Appendix Text 2. Details of studies’ scenarios**

Most study scenarios were based on planned interventions. Study scenarios were formulated as follows: Ghaffarzadegan had two “policy effect” scenarios: the first being status quo contact rate, and the second was “aggressive efforts to decrease contact rate by half what it would be otherwise” [41]. Haghdoost had a Basic scenario (no intervention) with only 10% isolation (we named it S0), and three intervention scenarios that had different levels of isolation: S1, Worst scenario, with minimum (25%) isolation; S2, Medium scenario, medium (32%) isolation; and S3, Best scenario, maximum (40%) isolation [27].

Mashayekhi had three scenarios. S1: Ideal scenario, serious distancing. Individuals reduce their social [physical] contacts to 20% of regular level, voluntarily or on a forced basis, after number of cases and deaths have increased, plus commitment to recommended personal hygiene, so that transmission rate reduces by 65%. S2: Medium scenario, not serious distancing. Individuals reduce their social [physical] contacts only to 20% of regular level, voluntarily, after number of cases and deaths have increased, and other settings are like scenario 1. S3: Worst scenario. Individuals reduce their social [physical] contacts only to 50% of regular level, voluntarily, after number of cases and deaths have increased, plus inadequate commitment to recommended personal hygiene, so that transmission rate reduces only by 40% (instead of 55%), and 60% of people do not care about the recommendations [28].

Moradi had four scenarios with Case Fatality Rate values of 0.3%, 0.5%, 1%, and 2% [42]. Saberi (web site) had 12 final scenarios, a combination of three options for number of cases and deaths to start with, and four options for the susceptible population size [21]. The three options for number of cases and deaths were as follows. Scenario 1, Best scenario, based on official reports, with correction factor of 1. Scenario 2, Medium scenario, based on official reports with correction factor of 5 (referring to the comment of the WHO Director of Emergency Operations - Dr. Rick Brennan - on country mission report) [54]. Scenario 3, Worst scenario, based on official reports with correction factor of 10 (after Russell [55], 2020-03-30), with 80 million susceptible population. It is notable that Russell estimated proportion of symptomatic cases reported in different countries using corrected case fatality rate (cCFR) estimates based on case and death timeseries data from the European Center for Disease Control (ECDC) for each country, whose values were 12% (95% confidence interval 9.9% - 22%) for Iran. Those estimates result in correction factor of 8.33 (4.55-10.10). The four options for the susceptible population size were 10, 30, 50, and 80 million.

Two studies used flight and passenger data to estimate the number of cumulative cases at the certain point in calendar time in Iran [40, 46]. The scenarios they used where about possible events before the time they conducted their studies. Tuite had six scenarios, from which we report the main, as well as the best and the worst ones (the lowest and highest estimates, for the sake of brevity) [46]. Their main scenario was based on travel destinations United Arab Emirates, Lebanon, and Canada. The best scenario used the same destinations with assumption of with no additional undetected cases. The worst scenario was based on destinations United Arab Emirates, Lebanon, Canada, and Syria. Zhuang had five scenarios with selected combinations of effective catchment population size (81800269 or 60000000), detection window duration (8 or 10 days), and “load factor” (70% or 90%), that was the “attendance rate for each aircraft” [47]. Muniz-Rodriguez predicted daily cases only for 11 days [37]. Zhan [40] predicted Cumulative cases for one day at national level for Iran (14450 infected individuals, its peak around March 22, 2020).

Appendix Table 1. Predictions of cumulative cases for the end of months one to six after the official epidemic start date (2020-02-19) and the latest date available in 2020

|  | Date1^(a)^ | 20-03-19 | 20-04-19 | 20-05-20 | 20-06-20 | 20-07-21 | 20-08-21 | Latest date |
| --- | --- | --- | --- | --- | --- | --- | --- | --- |
|  | Date 2 ^(b)^ | 98-12-29 | 99-01-31 | 99-02-31 | 99-03-31 | 99-04-31 | 99-05-31 | in 2020 ^(c)^ |
| - First Author, Outcome | S/M ^(d)^ | Value | Value | Value | Value | Value | Value | Value |
| - MOHME official via [4, 5] |  |  |  |  |  |  |  |  |
| Cumulative cases | N/A ^(e)^ | 18407 | 82211 | 126949 | 202584 | 278827 | 354764 | 534631 |
| - Ahmadi [44] |  |  |  |  |  |  |  |  |
| Cumulative cases | M1 ^(f)^ | 22379 | 60720 | ·· | ·· | ·· | ·· | ·· |
| Cumulative cases | M2 ^(g)^ | 23857 | ·· | ·· | ·· | ·· | ·· | ·· |
| Cumulative cases | M3 ^(h)^ | 18797 | ·· | ·· | ·· | ·· | ·· | ·· |
| - Al-Qaness [52] |  |  |  |  |  |  |  |  |
| Cumulative cases | M1 ^(i)^ | ·· | 20588 | ·· | ·· | ·· | ·· | ·· |
| - DELPHI [10] |  |  |  |  |  |  |  |  |
| Cumulative cases | S1 ^(j)^ | ·· | ·· | ·· | 144305 | ·· | ·· | ·· |
| - Ghaffarzadegan [41] |  |  |  |  |  |  |  |  |
| Cumulative cases | S1P1 ^(k)^ | 892009 | 1489201 | 2245140 | 2917927 | ·· | ·· | ·· |
| Cumulative cases | S1P2 ^(l)^ | 892009 | 1285097 | 1632829 | 1995680 | ·· | ·· | ·· |
| Cumulative cases | S2P1 ^(m)^ | 892009 | 1489201 | 2245140 | 2494600 | ·· | ·· | ·· |
| Cumulative cases | S2P2 ^(n)^ | 892009 | 1285097 | 1557235 | 1700864 | ·· | ·· | ·· |
| Cumulative cases | S3P1 ^(o)^ | 892009 | 1489201 | 2260259 | 2109071 | ·· | ·· | ·· |
| Cumulative cases | S3P2 ^(p)^ | 892009 | 1285097 | 1549676 | 1602592 | ·· | ·· | ·· |
| - Haghdoost [27] |  |  |  |  |  |  |  |  |
| Cumulative cases | S0 ^(q)^ | ·· | ·· | 2400000 | ·· | ·· | ·· | ·· |
| Cumulative cases | S1 ^(r)^ | 499054 | 953943 | 1160000 | ·· | ·· | ·· | ·· |
| Cumulative cases | S2 ^(s)^ | 499054 | 953943 | 951000 | ·· | ·· | ·· | ·· |
| Cumulative cases | S3 ^(t)^ | 437764 | 704230 | 812991 | ·· | ·· | ·· | ·· |
| - Hsiang [45] |  |  |  |  |  |  |  |  |
| Cumulative cases, mean | S1 ^(u)^ | 34858 | ·· | ·· | ·· | ·· | ·· | ·· |
| Cumulative cases, lower | S1 ^(u)^ | 20915 | ·· | ·· | ·· | ·· | ·· | ·· |
| Cumulative cases, upper | S1 ^(u)^ | 120557 | ·· | ·· | ·· | ·· | ·· | ·· |
| Cumulative cases, mean | S2 ^(v)^ | 2952519 | ·· | ·· | ·· | ·· | ·· | ·· |
| Cumulative cases, lower | S2 ^(v)^ | 817658 | ·· | ·· | ·· | ·· | ·· | ·· |
| Cumulative cases, upper | S2 ^(v)^ | 5152391 | ·· | ·· | ·· | ·· | ·· | ·· |
| - IHME [12] |  |  |  |  |  |  |  |  |
| Cumulative cases, mean | S2 ^(w)^ | 1196457 | 2071870 | 2656008 | 3883058 | 5876800 | 7229207 | 24127996 |
| Cumulative cases, lower | S2 ^(w)^ | 1042288 | 1850357 | 2396081 | 3521705 | 5400417 | 6657784 | 18741138 |
| Cumulative cases, upper | S2 ^(w)^ | 1364663 | 2310161 | 2933810 | 4266964 | 6382434 | 7834209 | 30946644 |
| - Imperial [13] |  |  |  |  |  |  |  |  |
| Cumulative cases, mean | S1 ^(x)^ | 466603 | 1012836 | 1333043 | 1997259 | 3149416 | 4054071 | 6086486 |
| Cumulative cases, lower | S1 ^(x)^ | 246658 | 569017 | 762173 | 1107328 | 1792418 | 2396384 | 3873699 |
| Cumulative cases, upper | S1 ^(x)^ | 771339 | 1660324 | 2158458 | 3089719 | 4531705 | 5745373 | 8080340 |
| Cumulative cases, mean | S2 ^(y)^ | 466603 | 1012836 | 1333043 | 1997259 | 3149416 | 4054071 | 7368437 |
| Cumulative cases, lower | S2 ^(y)^ | 246658 | 569017 | 762173 | 1107328 | 1792418 | 2396384 | 4452216 |
| Cumulative cases, upper | S2 ^(y)^ | 771339 | 1660324 | 2158458 | 3089719 | 4531705 | 5745373 | 10538086 |
| Cumulative cases, mean | S3 ^(z)^ | 466603 | 1012836 | 1333043 | 1997259 | 3149416 | 4054071 | 23261754 |
| Cumulative cases, lower | S3 ^(z)^ | 246658 | 569017 | 762173 | 1107328 | 1792418 | 2396384 | 10574316 |
| Cumulative cases, upper | S3 ^(z)^ | 771339 | 1660324 | 2158458 | 3089719 | 4531705 | 5745373 | 41475792 |
| Cumulative cases, mean | S4 ^(aa)^ | 451435 | 978749 | 1285670 | 1929910 | 3026744 | 3910937 | 5987188 |
| Cumulative cases, lower | S4 ^(aa)^ | 208290 | 470166 | 631108 | 969464 | 1651301 | 2212179 | 3588293 |
| Cumulative cases, upper | S4 ^(aa)^ | 526097 | 1149210 | 1519426 | 2272553 | 3547836 | 4559518 | 6827581 |
| Cumulative cases, mean | S5 ^(bb)^ | 451435 | 978749 | 1285670 | 1929910 | 3026744 | 3910937 | 7281363 |
| Cumulative cases, lower | S5 ^(bb)^ | 208290 | 470166 | 631108 | 969464 | 1651301 | 2212179 | 4180021 |
| Cumulative cases, upper | S5 ^(bb)^ | 713454 | 1572376 | 2049462 | 2923909 | 4375507 | 5440386 | 11205780 |
| Cumulative cases, mean | S6 ^(cc)^ | 451435 | 978749 | 1285670 | 1929910 | 3026744 | 3910937 | 23486980 |
| Cumulative cases, lower | S6 ^(cc)^ | 208290 | 470166 | 631108 | 969464 | 1651301 | 2212179 | 10478336 |
| Cumulative cases, upper | S6 ^(cc)^ | 713454 | 1572376 | 2049462 | 2923909 | 4375507 | 5440386 | 40835116 |
| - LANL [14] |  |  |  |  |  |  |  |  |
| Cumulative cases, median | S1 ^(dd)^ | 18407 | 82211 | 126949 | 202584 | 278827 | 354764 | 605238 |
| Cumulative cases, lower | S1 ^(dd)^ | 18407 | 82211 | 126949 | 202584 | 278827 | 354764 | 541458 |
| Cumulative cases, upper | S1 ^(dd)^ | 18407 | 82211 | 126949 | 202584 | 278827 | 354764 | 757094 |
| - Moghadami [36] |  |  |  |  |  |  |  |  |
| Cumulative cases, mean | S1 ^(dd)^ | 19514 | 52840 | ·· | ·· | ·· | ·· | ·· |
| Cumulative cases, lower | S1 ^(dd)^ | 19160 | 33226 | ·· | ·· | ·· | ·· | ·· |
| Cumulative cases, upper | S1 ^(dd)^ | 19973 | 72242 | ·· | ·· | ·· | ·· | ·· |
| - Moradi [42] |  |  |  |  |  |  |  |  |
| Cumulative cases | S1 ^(ee)^ | 2617251 | ·· | ·· | ·· | ·· | ·· | ·· |
| Cumulative cases | S2 ^(ff)^ | 1570351 | ·· | ·· | ·· | ·· | ·· | ·· |
| Cumulative cases | S3 ^(gg)^ | 785175 | ·· | ·· | ·· | ·· | ·· | ·· |
| Cumulative cases | S4 ^(hh)^ | 392587 | ·· | ·· | ·· | ·· | ·· | ·· |
| - Tuite [46] |  |  |  |  |  |  |  |  |
| Cumulative cases, mean | S1 ^(ii)^ | 1816 | ·· | ·· | ·· | ·· | ·· | ·· |
| Cumulative cases, lower | S1 ^(ii)^ | 405 | ·· | ·· | ·· | ·· | ·· | ·· |
| Cumulative cases, upper | S1 ^(ii)^ | 5270 | ·· | ·· | ·· | ·· | ·· | ·· |
| Cumulative cases, mean | S2 ^(jj)^ | 18307 | ·· | ·· | ·· | ·· | ·· | ·· |
| Cumulative cases, lower | S2 ^(jj)^ | 3713 | ·· | ·· | ·· | ·· | ·· | ·· |
| Cumulative cases, upper | S2 ^(jj)^ | 53333 | ·· | ·· | ·· | ·· | ·· | ·· |
| Cumulative cases, mean | S3 ^(kk)^ | 20965 | ·· | ·· | ·· | ·· | ·· | ·· |
| Cumulative cases, lower | S3 ^(kk)^ | 5728 | ·· | ·· | ·· | ·· | ·· | ·· |
| Cumulative cases, upper | S3 ^(kk)^ | 53737 | ·· | ·· | ·· | ·· | ·· | ·· |
| - Zhuang [47] |  |  |  |  |  |  |  |  |
| Cumulative cases, mean | S1 ^(ll)^ | 16533 | ·· | ·· | ·· | ·· | ·· | ·· |
| Cumulative cases, lower | S1 ^(ll)^ | 5925 | ·· | ·· | ·· | ·· | ·· | ·· |
| Cumulative cases, upper | S1 ^(ll)^ | 35538 | ·· | ·· | ·· | ·· | ·· | ·· |
| Cumulative cases, mean | S2 ^(mm)^ | 12125 | ·· | ·· | ·· | ·· | ·· | ·· |
| Cumulative cases, lower | S2 ^(mm)^ | 4345 | ·· | ·· | ·· | ·· | ·· | ·· |
| Cumulative cases, upper | S2 ^(mm)^ | 12145 | ·· | ·· | ·· | ·· | ·· | ·· |
| Cumulative cases, mean | S3 ^(nn)^ | 20667 | ·· | ·· | ·· | ·· | ·· | ·· |
| Cumulative cases, lower | S3 ^(nn)^ | 7408 | ·· | ·· | ·· | ·· | ·· | ·· |
| Cumulative cases, upper | S3 ^(nn)^ | 44424 | ·· | ·· | ·· | ·· | ·· | ·· |
| Cumulative cases, mean | S4 ^(oo)^ | 18368 | ·· | ·· | ·· | ·· | ·· | ·· |
| Cumulative cases, lower | S4 ^(oo)^ | 6583 | ·· | ·· | ·· | ·· | ·· | ·· |
| Cumulative cases, upper | S4 ^(oo)^ | 39482 | ·· | ·· | ·· | ·· | ·· | ·· |
| Cumulative cases, mean | S5 ^(pp)^ | 23627 | ·· | ·· | ·· | ·· | ·· | ·· |
| Cumulative cases, lower | S5 ^(pp)^ | 8472 | ·· | ·· | ·· | ·· | ·· | ·· |
| Cumulative cases, upper | S5 ^(pp)^ | 50780 | ·· | ·· | ·· | ·· | ·· | ·· |

(a) Date 1: Gregorian.

(b) Date 2: Hijri.

(c) Latest date in 2020: As of 2020-10-19 for MOHME official via [4, 5], 2020-12-31 for Imperial [13].

(d) S/M: Scenario / Model.

(e) N/A: Not Applicable.

(f) M1: Gompertz.

(g) M2: Von Bertalanffy growth.

(h) M3: Cubic Polynomial.

(i) M1: Adaptive Neuro-Fuzzy Inference System (ANFIS) enhanced with Genetic Algorithm (GA). They have 6 models: (1) Adaptive Neuro-Fuzzy Inference System (ANFIS) enhanced with Genetic Algorithm (GA), (2) Original Adaptive Neuro-Fuzzy Inference System (ANFIS), (3) Particle Swarm Optimizer(PSO), (4) Artificial Bee Colony (ABC), (5) Hybridized of Flower Pollination Algorithm and SALP Swarm Algorithm (SSAFPA), (6) Sine-Cosine Algorithm (SCA). The first one, Adaptive Neuro-Fuzzy Inference System (ANFIS) enhanced with Genetic Algorithm (GA), had the best accuracy performance.

(j) S1: Single scenario.

(k) S1P1: Seasonality conditions 1 (no effect or status quo) and Policy effect 1 (status quo contact rate). Estimates for 2020-03-19, the end of first month after the epidemic start date, are equal across the six scenarios.

(l) S1P2: Seasonality conditions 1 (no effect or status quo) and Policy effect 2 (aggressive efforts to decrease contact rate by half of what it would be otherwise).

(m) S2P1: Seasonality conditions 2 (moderate effect; infectivity of the virus decreases linearly from April 1st and halves by June 1st, then stays the same for the rest of the simulation) and Policy effect 1 (status quo contact rate).

(n) S2P2: Seasonality conditions 2 (moderate effect; infectivity of the virus decreases linearly from April 1st and halves by June 1st, then stays the same for the rest of the simulation) and Policy effect 2 (aggressive efforts to decrease contact rate by half of what it would be otherwise).

(o) S3P1: Seasonality conditions 3 (very strong mitigating effect; infectivity of the virus decreases from April 1st to a quarter of its base value by June 1st, then stays the same for the rest of the simulation) and Policy effect 1 (status quo contact rate).

(p) S3P2: Seasonality conditions 3 (very strong mitigating effect; infectivity of the virus decreases from April 1st to a quarter of its base value by June 1st, then stays the same for the rest of the simulation) and Policy effect 2 (aggressive efforts to decrease contact rate by half of what it would be otherwise).

(q) S0: Basic scenario (no intervention), only 10% isolation.

(r) S1: Worst scenario, minimum (25%) isolation.

(s) S2: Medium scenario, medium (32%) isolation.

(t) S3: Best scenario, maximum (40%) isolation.

(u) S1: Scenario Actual Policies.

(v) S2: Scenario No-policy.

(w) S2 Reference (Current): ‘Current projection’ scenario assumes social distancing mandates are re-imposed for 6 weeks whenever daily deaths reach 8 per million (0.8 per 100,000).

(x) S1: Additional 50% Reduction.

(y) S2: Maintain Status Quo.

(z) S3: Relax Interventions 50%.

(aa) S4: Surged Additional 50% Reduction.

(bb) S5: Surged Maintain Status Quo.

(cc) S6: Surged Relax Interventions 50%.

(dd) S1: Single scenario.

(ee) S1, Case Fatality Rate, 0.3%

(ff) S2, Case Fatality Rate, 0.5%

(gg) S3, Case Fatality Rate, 1%

(hh) S4, Case Fatality Rate, 2%

(ii) S1: Best scenario, United Arab Emirates, Lebanon, Canada (travels) with no additional undetected cases. Date: Late February 2020. Exact date not mentioned.

(jj) S2: Main scenario, United Arab Emirates, Lebanon, Canada (travels). Date: Late February 2020. Exact date not mentioned.

(kk) S3: Worst scenario, United Arab Emirates, Lebanon, Canada, Syria (travels). Date: Late February 2020. Exact date not mentioned.

(ll) S1: Baseline scenario. Effective catchment population 81800269; detection window, 10 days. Date: 2020-02-25.

(mm) S2: Smaller catchment scenario. Effective catchment population 60000000; detection window, 10 days. Date: 2020-02-25.

(nn) S3: Shorter detection window. Effective catchment population 81800269; detection window, 8 days. Date: 2020-02-25.

(oo) S4: 90% load factor. Effective catchment population 81800269; detection window, 10 days. Date: 2020-02-25.

(pp) S5: 70% load factors. Effective catchment population 81800269; detection window, 10 days. Date: 2020-02-25.

Appendix Table 2. Predictions of daily deaths at end of months one to six after the official epidemic start date (2020-02-19) and the latest date available in 2020

|  | Date 1 ^(a)^ | 20-03-19 | 20-04-19 | 20-05-20 | 20-06-20 | 20-07-21 | 20-08-21 | Latest date |
| --- | --- | --- | --- | --- | --- | --- | --- | --- |
|  | Date 2 ^(b)^ | 98-12-29 | 99-01-31 | 99-02-31 | 99-03-31 | 99-04-31 | 99-05-31 | in 2020 ^(c)^ |
| - First Author, Outcome | S/M ^(d)^ | Value | Value | Value | Value | Value | Value | Value |
| - MOHME official via[4, 5] |  |  |  |  |  |  |  |  |
| New deaths | N/A ^(e)^ | 149 | 87 | 64 | 115 | 229 | 112 | ·· |
| - DELPHI [10] |  |  |  |  |  |  |  |  |
| Detected deaths daily | S1 ^(f)^ | ·· | ·· | ·· | 12 | ·· | ·· | ·· |
| - Gu (YYG) [17] |  |  |  |  |  |  |  |  |
| Daily deaths, mean | S1 ^(f)^ | ·· | ·· | ·· | ·· | ·· | ·· | 189 |
| Daily deaths, mean | S1 ^(f)^ | ·· | ·· | ·· | ·· | ·· | ·· | 73 |
| Daily deaths, mean | S1 ^(f)^ | ·· | ·· | ·· | ·· | ·· | ·· | 387 |
| - IHME [12] |  |  |  |  |  |  |  |  |
| Daily deaths, mean ^(g)^ | S1 ^(h)^ | 137 | 94 | 54 | 113 | 211 | 136 | 210 |
| Daily deaths, lower ^(g)^ | S1 ^(h)^ | 137 | 94 | 54 | 113 | 211 | 136 | 72 |
| Daily deaths, upper ^(g)^ | S1 ^(h)^ | 137 | 94 | 54 | 113 | 211 | 136 | 382 |
| Daily deaths, mean ^(g)^ | S2 ^(i)^ | 137 | 94 | 54 | 113 | 211 | 136 | 605 |
| Daily deaths, lower ^(g)^ | S2 ^(i)^ | 137 | 94 | 54 | 113 | 211 | 136 | 382 |
| Daily deaths, upper ^(g)^ | S2 ^(i)^ | 137 | 94 | 54 | 113 | 211 | 136 | 908 |
| Daily deaths, mean ^(g)^ | S3 ^(j)^ | 137 | 94 | 54 | 113 | 211 | 136 | 1029 |
| Daily deaths, lower ^(g)^ | S3 ^(j)^ | 137 | 94 | 54 | 113 | 211 | 136 | 669 |
| Daily deaths, upper ^(g)^ | S3 ^(j)^ | 137 | 94 | 54 | 113 | 211 | 136 | 1456 |
| - Imperial [13] |  |  |  |  |  |  |  |  |
| Daily deaths, mean | S1 ^(k)^ | 70 | 69 | 46 | 98 | 166 | 119 | 0 |
| Daily deaths, lower | S1 ^(k)^ | 33 | 38 | 22 | 48 | 98 | 80 | 0 |
| Daily deaths, upper | S1 ^(k)^ | 116 | 111 | 79 | 137 | 235 | 164 | 2 |
| Daily deaths, mean | S2 ^(l)^ | 70 | 69 | 46 | 98 | 166 | 119 | 56 |
| Daily deaths, lower | S2 ^(l)^ | 33 | 38 | 22 | 48 | 98 | 80 | 5 |
| Daily deaths, upper | S2 ^(l)^ | 116 | 111 | 79 | 137 | 235 | 164 | 166 |
| Daily deaths, mean | S3 ^(m)^ | 70 | 69 | 46 | 98 | 166 | 119 | 2396 |
| Daily deaths, lower | S3 ^(m)^ | 33 | 38 | 22 | 48 | 98 | 80 | 484 |
| Daily deaths, upper | S3 ^(m)^ | 116 | 111 | 79 | 137 | 235 | 164 | 3984 |
| Daily deaths, mean | S4 ^(n)^ | 68 | 66 | 44 | 96 | 157 | 116 | 0 |
| Daily deaths, lower | S4 ^(n)^ | 33 | 30 | 22 | 50 | 92 | 73 | 0 |
| Daily deaths, upper | S4 ^(n)^ | 106 | 113 | 78 | 149 | 228 | 164 | 2 |
| Daily deaths, mean | S5 ^(o)^ | 68 | 66 | 44 | 96 | 157 | 116 | 55 |
| Daily deaths, lower | S5 ^(o)^ | 33 | 30 | 22 | 50 | 92 | 73 | 6 |
| Daily deaths, upper | S5 ^(o)^ | 106 | 113 | 78 | 149 | 228 | 164 | 185 |
| Daily deaths, mean | S6 ^(p)^ | 68 | 66 | 44 | 96 | 157 | 116 | 1,612 |
| Daily deaths, lower | S6 ^(p)^ | 33 | 30 | 22 | 50 | 92 | 73 | 412 |
| Daily deaths, upper | S6 ^(p)^ | 106 | 113 | 78 | 149 | 228 | 164 | 2871 |
| - LANL [14] |  |  |  |  |  |  |  |  |
| Daily deaths, median | S1 ^(f)^ | 149 | 87 | 64 | 115 | 229 | 112 | 52 |
| Daily deaths, lower | S1 ^(f)^ | 149 | 87 | 64 | 115 | 229 | 112 | 8 |
| Daily deaths, upper | S1 ^(f)^ | 149 | 87 | 64 | 115 | 229 | 112 | 278 |
| - Mashayekhi [28] |  |  |  |  |  |  |  |  |
| Daily deaths | S1 ^(q)^ | 224 | 125 | 10 | 5 | ·· | ·· | ·· |
| Daily deaths | S2 ^(r)^ | 245 | 1194 | 658 | 403 | 239 | 139 | ·· |
| Daily deaths | S3 ^(s)^ | 176 | 7839 | 27704 | 44934 | 29270 | 7782 | ·· |
| Rahimi Rise [29] |  |  |  |  |  |  |  |  |
| Daily deaths | S1 ^(t)^ | 12611 | 6001 | 6204 | 4374 | 3459 | ·· | ·· |
| Daily deaths | S2 ^(u)^ | 24560 | 11289 | 9916 | 7018 | 5035 | ·· | ·· |

(a) Date 1: Gregorian.

(b) Date 2: Hijri.

(c) Latest date in 2020: 2020-11-01for Gu (YYG) [17], 2020-12-31 for IHME [12] and Imperial [13], and 2020-11-28 for LANL [14].

(d) S/M: Scenario / Model.

(e) N/A: Not Applicable.

(f) S1: Single scenario.

(g) Smoothed estimates.

(h) S1 Best (Masks): ‘Universal Masks’ scenario reflects 95% mask usage in public in every location.

(i) S2 Reference (Current): ‘Current projection’ scenario assumes social distancing mandates are re-imposed for 6 weeks whenever daily deaths reach 8 per million (0.8 per 100,000).

(j) S3 Worse (Easing): ‘Mandates easing’ scenario reflects continued easing of social distancing mandates, and mandates are not re-imposed.

(k) S1: Additional 50% Reduction.

(l) S2: Maintain Status Quo.

(m) S3: Relax Interventions 50%.

(n) S4: Surged Additional 50% Reduction.

(o) S5: Surged Maintain Status Quo.

(p) S6: Surged Relax Interventions 50%.

(q) S1: Serious distancing.

(r) S2: Not serious distancing.

(s) S3: Worse than Scenario 2.

(t) S1: Interventions.

(u) S2: No interventions.

Appendix Table 3. Predictions of daily cases for the end of months one to six after the official epidemic start date (2020-02-19) and the latest date available in 2020

|  | Date 1 ^(a)^ | 20-03-19 | 20-04-19 | 20-05-20 | 20-06-20 | 20-07-21 | 20-08-21 | Latest date |
| --- | --- | --- | --- | --- | --- | --- | --- | --- |
|  | Date 2 ^(b)^ | 98-12-29 | 99-01-31 | 99-02-31 | 99-03-31 | 99-04-31 | 99-05-31 | in 2020 ^(c)^ |
| - First Author, Outcome | S/M ^(d)^ | Value | Value | Value | Value | Value | Value | Value |
| ***Incident Daily Cases*** |  |  |  |  |  |  |  |  |
| - MOHME official via[4, 5] | N/A ^(e)^ | 1046 | 1343 | 2346 | 2322 | 2625 | 2206 | 4251 |
|  |  |  |  |  |  |  |  |  |
| - Ayyoubzadeh [84] |  |  |  |  |  |  |  |  |
| Normalized Daily cases | M1 ^(f)^ | 92 | ·· | ·· | ·· | ·· | ·· | ·· |
| Normalized Daily cases | M2 ^(g)^ | 86 | ·· | ·· | ·· | ·· | ·· | ·· |
| - DELPHI [10] |  |  |  |  |  |  |  |  |
| Daily cases | S1 ^(h)^ | ·· | ·· | ·· | 211 | ·· | ·· | ·· |
| - Gu (YYG) [17] |  |  |  |  |  |  |  |  |
| Daily incident cases, mean | S1 ^(h)^ | 35251 | 24101 | 44963 | 85417 | 99500 | 91952 | 126637 |
| Daily incident cases, lower | S1 ^(h)^ | 13279 | 8693 | 23653 | 49804 | 69771 | 61529 | 24229 |
| Daily incident cases, upper | S1 ^(h)^ | 61369 | 44894 | 68707 | 138892 | 135612 | 131357 | 363726 |
| - Haghdoost [27] |  |  |  |  |  |  |  |  |
| New cases | S1 ^(i)^ | 12454 | 13460 | 7859 | ·· | ·· | ·· | ·· |
| New cases | S2 ^(j)^ | 11993 | 9716 | 4254 | ·· | ·· | ·· | ·· |
| New cases | S3 ^(k)^ | 11427 | 6934 | 2272 | ·· | ·· | ·· | ·· |
| - IHME [12] |  |  |  |  |  |  |  |  |
| Daily cases, mean | S1 ^(l)^ | ·· | ·· | ·· | ·· | ·· | ·· | 73181 |
| Daily cases, lower | S1 ^(l)^ | ·· | ·· | ·· | ·· | ·· | ·· | 20144 |
| Daily cases, upper | S1 ^(l)^ | ·· | ·· | ·· | ·· | ·· | ·· | 137549 |
| Daily cases, mean | S2 ^(m)^ | 40145 | 19069 | 23709 | 55687 | 58724 | 39444 | 103283 |
| Daily cases, lower | S2 ^(m)^ | 37429 | 17811 | 21532 | 5802 | 54,567 | 36540 | 60240 |
| Daily cases, upper | S2 ^(m)^ | 42991 | 20381 | 25947 | 59799 | 62790 | 42701 | 215305 |
| Daily cases, mean | S3 ^(n)^ | ·· | ·· | ·· | ·· | ·· | ·· | 330112 |
| Daily cases, lower | S3 ^(n)^ | ·· | ·· | ·· | ·· | ·· | ·· | 232989 |
| Daily cases, upper | S3 ^(n)^ | ·· | ·· | ·· | ·· | ·· | ·· | 435400 |
| - Imperial [13] |  |  |  |  |  |  |  |  |
| Daily cases, mean | S1 ^(o)^ | 28406 | 9240 | 12323 | 31110 | 46823 | 24554 | 6 |
| Daily cases, lower | S1 ^(o)^ | 15380 | 5503 | 6497 | 16495 | 29115 | 17007 | 0 |
| Daily cases, upper | S1 ^(o)^ | 46666 | 14903 | 19728 | 43929 | 65375 | 32640 | 18 |
| Daily cases, mean | S2 ^(p)^ | 28406 | 9240 | 12323 | 31110 | 46823 | 24554 | 9915 |
| Daily cases, lower | S2 ^(p)^ | 15380 | 5503 | 6497 | 16495 | 29115 | 17007 | 943 |
| Daily cases, upper | S2 ^(p)^ | 46666 | 14903 | 19728 | 43929 | 65375 | 32640 | 35532 |
| Daily cases, mean | S3 ^(q)^ | 28406 | 9240 | 12323 | 31110 | 46823 | 24554 | 331842 |
| Daily cases, lower | S3 ^(q)^ | 15380 | 5503 | 6497 | 16495 | 29115 | 17007 | 104036 |
| Daily cases, upper | S3 ^(q)^ | 46666 | 14903 | 19728 | 43929 | 65375 | 32640 | 486745 |
| Daily cases, mean | S4 ^(r)^ | 27448 | 8867 | 11939 | 29731 | 44829 | 24805 | 6 |
| Daily cases, lower | S4 ^(r)^ | 12980 | 4415 | 5823 | 16503 | 27445 | 1014 | 0 |
| Daily cases, upper | S4 ^(r)^ | 43892 | 14933 | 17615 | 41329 | 65951 | 31493 | 25 |
| Daily cases, mean | S5 ^(s)^ | 27448 | 8867 | 11939 | 29731 | 44829 | 24805 | 9700 |
| Daily cases, lower | S5 ^(s)^ | 12980 | 4415 | 5823 | 16503 | 27445 | 1,014 | 1029 |
| Daily cases, upper | S5 ^(s)^ | 43892 | 14933 | 17615 | 41329 | 65951 | 31493 | 36023 |
| Daily cases, mean | S6 ^(t)^ | 27448 | 8867 | 11939 | 29731 | 44829 | 24805 | 335941 |
| Daily cases, lower | S6 ^(t)^ | 12980 | 4415 | 5823 | 16503 | 27445 | 17014 | 111706 |
| Daily cases, upper | S6 ^(t)^ | 43892 | 14933 | 17615 | 41329 | 65951 | 31493 | 471837 |
| - LANL [14] |  |  |  |  |  |  |  |  |
| Daily cases, median | S1 ^(h)^ | 1046 | 1343 | 2346 | 2322 | 2625 | 2206 | 963 |
| Daily cases, lower | S1 ^(h)^ | 1046 | 1343 | 2346 | 2322 | 2625 | 2206 | 156 |
| Daily cases, upper | S1 ^(h)^ | 1046 | 1343 | 2346 | 2322 | 2625 | 2206 | 5074 |
| - Moftakhar [87] |  |  |  |  |  |  |  |  |
| Daily cases, mean | S1 ^(h)^ | 1046 | 3461 | ·· | ·· | ·· | ·· | ·· |
| Daily cases, lower | S1 ^(h)^ | ·· | 573 | ·· | ·· | ·· | ·· | ·· |
| Daily cases, upper | S1 ^(h)^ | ·· | 8786 | ·· | ·· | ·· | ·· | ·· |
| - Rafieenasab [51] |  |  |  |  |  |  |  |  |
| Daily cases | S2 ^(u)^ | 15793 | 2037 | ·· | ·· | ·· | ·· | ·· |
| Daily cases | S4 ^(v)^ | 4679 | 2948 | 207 | ·· | ·· | ·· | ·· |
| - Rahimi Rise [29] |  |  |  |  |  |  |  |  |
| Daily cases | S1 ^(w)^ | 57031 | 67723 | 62181 | 48520 | 38919 | ·· | ·· |
| Daily cases | S2 ^(x)^ | 171479 | 216262 | 175818 | 137806 | 102232 | ·· | ·· |
| - Shen [43] |  |  |  |  |  |  |  |  |
| Daily cases | S1 ^(y)^ | 4 | 653 | ·· | ·· | ·· | ·· | ·· |
| Daily cases | S2 ^(z)^ | 12 | 1152 | ·· | ·· | ·· | ·· | ·· |
| Daily cases | S3 ^(aa)^ | 1410 | 1027 | ·· | ·· | ·· | ·· | ·· |
| - Thu [48] |  |  |  |  |  |  |  |  |
| Daily cases | M1 ^(bb)^ | 1354 | 1308 | ·· | ·· | ·· | ·· | ·· |
| Daily cases | M1 ^(cc)^ | 122 | 93 | ·· | ·· | ·· | ·· | ·· |
| - Zareie [50] |  |  |  |  |  |  |  |  |
| Daily cases | S1 ^(h)^ | 1087 | 650 | ·· | ·· | ·· | ·· | ·· |
| ***Prevalent Daily Cases*** |  |  |  |  |  |  |  |  |
| - Ghaffarzadegan [41] |  |  |  |  |  |  |  |  |
| Current cases | S1P1 ^(dd)^ | 491126 | 277846 | 324026 | 314798 | ·· | ·· | ·· |
| Current cases | S1P2 ^(ee)^ | 491126 | 150818 | 157809 | 167500 | ·· | ·· | ·· |
| Current cases | S2P1 ^(ff)^ | 491126 | 277846 | 261864 | 158041 | ·· | ·· | ·· |
| Current cases | S2P2 ^(gg)^ | 491126 | 150818 | 119971 | 49935 | ·· | ·· | ·· |
| Current cases | S3P1 ^(hh)^ | 491126 | 277846 | 228080 | 63448 | ·· | ·· | ·· |
| Current cases | S3P2 ^(ii)^ | 491126 | 150818 | 102404 | 20205 | ·· | ·· | ·· |
| - Gu (YYG) [17] |  |  |  |  |  |  |  |  |
| Daily prevalent cases, mean | S1 ^(h)^ | 610183 | 362487 | 574001 | 1150831 | 1472947 | 1428598 | 1729160 |
| Daily prevalent cases, lower | S1 ^(h)^ | 216268 | 131352 | 285590 | 658556 | 1004842 | 998104 | 412656 |
| Daily prevalent cases, upper | S1 ^(h)^ | 1143720 | 668487 | 902802 | 1869856 | 2064466 | 1993535 | 4620216 |
| - Haghdoost [27] |  |  |  |  |  |  |  |  |
| Maximum cases per day | S0 ^(jj)^ | ·· | ·· | 1600000 | ·· | ·· | ·· | ·· |
| Maximum cases per day | S1 ^(i)^ | ·· | ·· | 91300 | ·· | ·· | ·· | ·· |
| Maximum cases per day | S2 ^(j)^ | ·· | ·· | 92100 | ·· | ·· | ·· | ·· |
| Maximum cases per day | S3 ^(k)^ | ·· | ·· | 9150 | ·· | ·· | ·· | ·· |
| - Mashayekhi [28] |  |  |  |  |  |  |  |  |
| Daily [symptomatic?] | S1 ^(kk)^ | 36946 | 370 | 370 | 655 | ·· | ·· | ·· |
| Daily asymptomatic case | S1 ^(kk)^ | 148344 | 6698 | 370 | 655 | ·· | ·· | ·· |
| Daily [symptomatic?] ^(t)^ | S2 ^(ll)^ | 81383 | 281442 | 192199 | 117139 | 69563 | 40307 | ·· |
| Daily asymptomatic cases ^(v)^ | S2 ^(ll)^ | 206678 | 183333 | 115662 | 69858 | 40603 | 24054 | ·· |
| Daily [symptomatic?] ^(w)^ | S3 ^(mm)^ | 826260 | 101577 | 2228704 | 3506023 | 2332975 | 805405 | ·· |
| Daily asymptomatic case ^(w)^ | S3 ^(mm)^ | 226702 | 760221 | 1889823 | 2311252 | 1142548 | 330104 | ·· |
| - Saberi (web site) [21] |  |  |  |  |  |  |  |  |
| Daily active cases | S1P10 ^(nn)^ | 28999 | 144333 | 1125843 | 2168910 | 1072138 | 331782 | ·· |
| Daily active cases | S1P30 ^(oo)^ | 74594 | 147681 | 1385549 | 5970776 | 5129813 | 1814997 | ·· |
| Daily active cases | S1P50 ^(pp)^ | 39758 | 229167 | 1581030 | 8407606 | 9655053 | 3914672 | ·· |
| Daily active cases | S1P80 ^(qq)^ | 17394 | 83007 | 829045 | 11116260 | 17146193 | 7887213 | ·· |
| Daily active cases | S2P10 ^(rr)^ | 97629 | 657612 | 2155808 | 1450279 | 483272 | 151105 | ·· |
| Daily active cases | S2P30 ^(ss)^ | 113556 | 712576 | 4369618 | 6238980 | 2685992 | 803934 | ·· |
| Daily active cases | S2P50 ^(tt)^ | 166153 | 826643 | 5535232 | 10922721 | 5462056 | 1802843 | ·· |
| Daily active cases | S2P80 ^(uu)^ | 87135 | 765505 | 6266482 | 17479235 | 10561303 | 3484396 | ·· |
| Daily active cases | S3P10 ^(vv)^ | 155497 | 1135577 | 2215204 | 1071274 | 330704 | 64927 | ·· |
| Daily active cases | S3P30 ^(ww)^ | 166231 | 1277903 | 5910654 | 5216125 | 1905813 | 555933 | ·· |
| Daily active cases | S3P50 ^(xx)^ | 284459 | 1472165 | 8276968 | 9809578 | 4007948 | 1289460 | ·· |
| Daily active cases | S3P80 ^(yy)^ | 186313 | 1467698 | 10125068 | 17115184 | 7863266 | 2463785 | ·· |

(a) Date 1: Gregorian.

(b) Date 2: Hijri.

(c) Latest date in 2020: As of 2020-10-19 for MOHME Official via [4, 5], 2020-03-18 for Ayyoubzadeh [84], 2020-11-01 for DELPHI [10], 2020-12-31 for IHME [12] and Imperial [13], 2020-11-28 for LANL [14], and 2020-11-01 for Gu (YYG) [17].

(d) S/M: Scenario / Model.

(e) N/A: Not Applicable.

(f) M1: Logistic regression.

(g) M2: Long Short-Term Memory (LSTM).

(h) S1: Single scenario.

(i) S1: Worst scenario, minimum (25%) isolation.

(j) S2: Medium scenario, medium (32%) isolation.

(k) S3: Best scenario, maximum (40%) isolation.

(l) S1 Best (Masks): ‘Universal Masks’ scenario reflects 95% mask usage in public in every location.

(m) S2 Reference (Current): ‘Current projection’ scenario assumes social distancing mandates are re-imposed for 6 weeks whenever daily deaths reach 8 per million (0.8 per 100,000).

(n) S3 Worse (Easing): ‘Mandates easing’ scenario reflects continued easing of social distancing mandates, and mandates are not re-imposed.

(o) S1: Additional 50% Reduction.

(p) S2: Maintain Status Quo.

(q) S3: Relax Interventions 50%.

(r) S4: Surged Additional 50% Reduction.

(s) S5: Surged Maintain Status Quo.

(t) S6: Surged Relax Interventions 50%.

(u) S2: Based on SIR model.

(v) S4: Approximated.

(w) S1: Interventions.

(x) S2: No interventions.

(y) S1, based on cut-off 1, 2020-03-13.

(z) S2, based on cut-off 2, 2020-03-21.

(aa) S3, based on cut-off 3, 2020-04-09.

(bb) M1: Linear growth rate, equation 1.

(cc) M2: Linear growth rate, equation 2, with a decline rate of 100%.

(dd) S1P1: Seasonality conditions 1 (no effect or status quo) and Policy effect 1 (status quo contact rate). Estimates for 2020-03-19, the end of first month after the epidemic start date, are equal across the six scenarios.

(ee) S1P2: Seasonality conditions 1 (no effect or status quo) and Policy effect 2 (aggressive efforts to decrease contact rate by half of what it would be otherwise).

(ff) S2P1: Seasonality conditions 2 (moderate effect; infectivity of the virus decreases linearly from April 1st and halves by June 1st, then stays the same for the rest of the simulation) and Policy effect 1 (status quo contact rate).

(gg) S2P2: Seasonality conditions 2 (moderate effect; infectivity of the virus decreases linearly from April 1st and halves by June 1st, then stays the same for the rest of the simulation) and Policy effect 2 (aggressive efforts to decrease contact rate by half of what it would be otherwise).

(hh) S3P1: Seasonality conditions 3 (very strong mitigating effect; infectivity of the virus decreases from April 1st to a quarter of its base value by June 1st, then stays the same for the rest of the simulation) and Policy effect 1 (status quo contact rate).

(ii) S3P2: Seasonality conditions 3 (very strong mitigating effect; infectivity of the virus decreases from April 1st to a quarter of its base value by June 1st, then stays the same for the rest of the simulation) and Policy effect 2 (aggressive efforts to decrease contact rate by half of what it would be otherwise).

(jj) S0: Basic scenario (no intervention), only 10% isolation.

(kk) S1: Serious distancing.

(ll) S2: Not serious distancing.

(mm) S3: Worse than Scenario 2.

(nn) S1P10: Scenario 1 (Best scenario, based on official reports with correction factor of 1) with 10 million susceptible population.

(oo) S1P30:Scenario 1 (Best scenario, based on official reports with correction factor of 1) with 30 million susceptible population.

(pp) S1P50: Scenario 1 (Best scenario, based on official reports with correction factor of 1) with 50 million susceptible population.

(qq) S1P80: Scenario 1 (Best scenario, based on official reports with correction factor of 1) with 80 million susceptible population.

(rr) S2P10: Scenario 2 (Medium scenario, based on official reports with correction factor of 5 (after Dr. Rick Brennan, Director of Emergency Operations, World Health Organization [54]) with 10 million susceptible population.

(ss) S2P30: Scenario 2 (Medium scenario, based on official reports with correction factor of 5 (after Dr. Rick Brennan, Director of Emergency Operations, World Health Organization [54]) with 30 million susceptible population.

(tt) S2P50: Scenario 2 (Medium scenario, based on official reports with correction factor of 5 (after Dr. Rick Brennan, Director of Emergency Operations, World Health Organization [54]) with 50 million susceptible population.

(uu) S2P80: Scenario 2 (Medium scenario, based on official reports with correction factor of 5 (after Dr. Rick Brennan, Director of Emergency Operations, World Health Organization [54]) with 80 million susceptible population.

(vv) S3P10: Scenario 3 (Worst scenario, based on official reports with correction factor of 10 (after Russell [55]) with 80 million susceptible population.

(ww) S3P30: Scenario 3 (Worst scenario, based on official reports with correction factor of 10 (after Russell [55]) with 30 million susceptible population.

(xx) S3P50: Scenario 3 (Worst scenario, based on official reports with correction factor of 10 (after Russell [55]) with 50 million susceptible population.

(yy) S3P80: Scenario 3 (Worst scenario, based on official reports with correction factor of 10 (after Russell [55]) with 10 million susceptible population.

Appendix Table 4. Predictions of epidemic peak dates and values of outcomes *

| Study 1st author | Scenario / model, peak number | Outcome | Value | Date |
| --- | --- | --- | --- | --- |
|  |  | ***Daily Deaths*** |  |  |
| MOHME official via [4, 5] | N/A ^(a)^ 1st peak | Daily deaths | 158 | 20-04-04 |
| MOHME official via [4, 5] | N/A ^(a)^ 2nd peak | Daily deaths | 235 | 20-07-28 |
| MOHME official via [4, 5] | N/A ^(a)^ 3rd peak ^(b)^ | Daily deaths | 337 | 20-10-19 |
| IHME [12] | S1 ^(c)^ 4th peak | Daily deaths, mean | 233 | 20-10-24 |
| IHME [12] | S1 ^(c)^ 4th peak | Daily deaths, lower | .. | .. |
| IHME [12] | S1 ^(c)^ 4th peak | Daily deaths, upper | 436 | 21-01-28 |
| IHME [12] | S2 ^(d)^ 4th peak | Daily deaths, mean | 785 | 20-12-17 |
| IHME [12] | S2 ^(d)^ 4th peak | Daily deaths, lower | 551 | 20-12-19 |
| IHME [12] | S2 ^(d)^ 4th peak | Daily deaths, upper | 1044 | 20-12-12 |
| IHME [12] | S3 ^(e)^ 4th peak | Daily deaths, mean | 1067 | 21-01-09 |
| IHME [12] | S3 ^(e)^ 4th peak | Daily deaths, lower | 728 | 21-01-15 |
| IHME [12] | S3 ^(e)^ 4th peak | Daily deaths, upper | 1477 | 21-01-05 |
| Imperial [13] | S1 ^(f)^ 3rd peak | Daily deaths, mean | 184 | 20-09-26 |
| Imperial [13] | S1 ^(f)^ 3rd peak | Daily deaths, lower | 134 | 20-09-29 |
| Imperial [13] | S1 ^(f)^ 3rd peak | Daily deaths, upper | 263 | 20-10-07 |
| Imperial [13] | S2 ^(g)^ 3rd peak | Daily deaths, mean | 184 | 20-09-26 |
| Imperial [13] | S2 ^(g)^ 3rd peak | Daily deaths, lower | 134 | 20-09-29 |
| Imperial [13] | S2 ^(g)^ 3rd peak | Daily deaths, upper | 270 | 20-10-27 |
| Imperial [13] | S3 ^(h)^ 3rd peak | Daily deaths, mean | 2410 | 20-12-28 |
| Imperial [13] | S3 ^(h)^ 3rd peak | Daily deaths, lower | 525 | 21-01-02 |
| Imperial [13] | S3 ^(h)^ 3rd peak | Daily deaths, upper | 4968 | 20-12-11 |
| Imperial [13] | S4 ^(i)^ 3rd peak | Daily deaths, mean | 186 | 20-09-28 |
| Imperial [13] | S4 ^(i)^ 3rd peak | Daily deaths, lower | 140 | 20-09-24 |
| Imperial [13] | S4 ^(i)^ 3rd peak | Daily deaths, upper | 294 | 20-10-10 |
| Imperial [13] | S5 ^(j)^ 3rd peak | Daily deaths, mean | 186 | 20-09-28 |
| Imperial [13] | S5 ^(j)^ 3rd peak | Daily deaths, lower | 140 | 20-09-24 |
| Imperial [13] | S5 ^(j)^ 3rd peak | Daily deaths, upper | 295 | 20-10-16 |
| Imperial [13] | S6 ^(k)^ 3rd peak | Daily deaths, mean | 1622 | 21-01-02 |
| Imperial [13] | S6 ^(k)^ 3rd peak | Daily deaths, lower | 440 | 21-01-01 |
| Imperial [13] | S6 ^(k)^ 3rd peak | Daily deaths, upper | 3315 | 20-12-17 |
| LANL [14] | S1 ^(l)^ 1st peak | Daily deaths, median | 81 | 20-06-01 |
| LANL [14] | S1 ^(l)^ 1st peak | Daily deaths, lower | 81 | 20-06-01 |
| LANL [14] | S1 ^(l)^ 1st peak | Daily deaths, upper | 81 | 20-06-01 |
| LANL [14] | S1 ^(l)^ 2nd peak | Daily deaths, median | 235 | 20-07-28 |
| LANL [14] | S1 ^(l)^ 2nd peak | Daily deaths, lower | 235 | 20-07-28 |
| LANL [14] | S1 ^(l)^ 2nd peak | Daily deaths, upper | 235 | 20-07-28 |
| LANL [14] | S1 ^(l)^ 3rd peak | Daily deaths, median | .. | .. |
| LANL [14] | S1 ^(l)^ 3rd peak | Daily deaths, lower | .. | .. |
| LANL [14] | S1 ^(l)^ 3rd peak | Daily deaths, upper | 372 | 20-10-25 |
| Mashayekhi [28] | S1 ^(m)^ Single peak | Daily deaths | 443 | 20-03-27 |
| Mashayekhi [28] | S2 ^(n)^ Single peak | Daily deaths | 1283 | 20-04-10 |
| Mashayekhi [28] | S3 ^(o)^ Single peak | Daily deaths | 44934 | 20-06-20 |
| Mashayekhi [28] | S1 ^(p)^ Highest peak | Daily deaths | 13831 | 20-03-14 |
| Mashayekhi [28] | S2 ^(q)^ Highest peak | Daily deaths | 27559 | 20-03-14 |
|  |  | ***Incident Daily Cases*** |  |  |
| MOHME official via [4, 5] | N/A ^(a)^ 1st peak | Daily cases | 3186 | 20-03-30 |
| MOHME official via [4, 5] | N/A ^(a)^ 2nd peak | Daily cases | 3574 | 20-06-04 |
| MOHME official via [4, 5] | N/A ^(a)^ 3rd peak ^(b)^ | Daily cases | 4830 | 20-10-14 |
| Ayyoubzadeh [84] | S1 ^(r)^ highest peak | Normalized daily cases | 93 | 20-03-16 |
| Ayyoubzadeh [84] | S2 ^(s)^ highest peak | Normalized daily cases | 86 | 20-03-16 |
| Gu (YYG) [17] | S1 ^(l)^ 1st peak | New cases, mean | 44772 | 20-03-05 |
| Gu (YYG) [17] | S1 ^(l)^ 1st peak | New cases, lower | 15207 | 20-03-05 |
| Gu (YYG) [17] | S1 ^(l)^ 1st peak | New cases, upper | 86393 | 20-03-05 |
| Gu (YYG) [17] | S1 ^(l)^ 2nd peak | New cases, mean | 99692 | 20-07-25 |
| Gu (YYG) [17] | S1 ^(l)^ 2nd peak | New cases, lower | 71102 | 20-07-25 |
| Gu (YYG) [17] | S1 ^(l)^ 2nd peak | New cases, upper | 133432 | 20-07-25 |
| Haghdoost [27] | S1 ^(t)^ 1st peak | New cases | 13512 | 20-03-02 |
| Haghdoost [27] | S1 ^(t)^ 2nd peak | New cases | 14133 | 20-03-11 |
| Haghdoost [27] | S1 ^(t)^ 3rd peak | New cases | 15172 | 20-04-10 |
| Haghdoost [27] | S2 ^(u)^ 1st peak | New cases | 13514 | 20-03-02 |
| Haghdoost [27] | S2 ^(u)^ 2nd peak | New cases | 14203 | 20-03-11 |
| Haghdoost [27] | S2 ^(u)^ 3rd peak | New cases | 11771 | 20-04-10 |
| Haghdoost [27] | S3 ^(v)^ 1st peak | New cases | 13689 | 20-03-02 |
| Haghdoost [27] | S3 ^(v)^ 2nd peak | New cases | 14272 | 20-03-11 |
| Haghdoost [27] | S3 ^(v)^ 3rd peak | New cases | 9223 | 20-04-10 |
| IHME [12] | S1 ^(c)^ 4th peak | Daily infections, mean | 76,020 | 20-10-06 |
| IHME [12] | S1 ^(c)^ 4th peak | Daily infections, lower | .. | .. |
| IHME [12] | S1 ^(c)^ 4th peak | Daily infections, upper | 139733 | 21-01-08 |
| IHME [12] | S2 ^(d)^ 4th peak | Daily infections, mean | 254850 | 20-11-29 |
| IHME [12] | S2 ^(d)^ 4th peak | Daily infections, lower | 179073 | 20-12-01 |
| IHME [12] | S2 ^(d)^ 4th peak | Daily infections, upper | 335289 | 20-11-23 |
| IHME [12] | S3 ^(e)^ 4th peak | Daily infections, mean | 342539 | 20-12-22 |
| IHME [12] | S3 ^(e)^ 4th peak | Daily infections, lower | 233422 | 20-12-28 |
| IHME [12] | S3 ^(e)^ 4th peak | Daily infections, upper | 472646 | 20-12-19 |
| Imperial [13] | S1 ^(f)^ 3rd peak | Daily infections, mean | 44292 | 20-09-11 |
| Imperial [13] | S1 ^(f)^ 3rd peak | Daily infections, lower | 33169 | 20-09-11 |
| Imperial [13] | S1 ^(f)^ 3rd peak | Daily infections, upper | 56759 | 20-09-25 |
| Imperial [13] | S2 ^(g)^ 3rd peak | Daily infections, mean | 44292 | 20-09-11 |
| Imperial [13] | S2 ^(g)^ 3rd peak | Daily infections, lower | 33169 | 20-09-11 |
| Imperial [13] | S2 ^(g)^ 3rd peak | Daily infections, upper | 56759 | 20-09-25 |
| Imperial [13] | S3 ^(h)^ 3rd peak | Daily infections, mean | 348479 | 20-12-20 |
| Imperial [13] | S3 ^(h)^ 3rd peak | Daily infections, lower | 107873 | 21-01-03 |
| Imperial [13] | S3 ^(h)^ 3rd peak | Daily infections, upper | 691530 | 20-12-04 |
| Imperial [13] | S4 ^(i)^ 3rd peak | Daily infections, mean | 45218 | 20-09-11 |
| Imperial [13] | S4 ^(i)^ 3rd peak | Daily infections, lower | 31711 | 20-09-11 |
| Imperial [13] | S4 ^(i)^ 3rd peak | Daily infections, upper | 61982 | 20-09-25 |
| Imperial [13] | S5 ^(j)^ 3rd peak | Daily infections, mean | 45218 | 20-09-11 |
| Imperial [13] | S5 ^(j)^ 3rd peak | Daily infections, lower | 31711 | 20-09-11 |
| Imperial [13] | S5 ^(j)^ 3rd peak | Daily infections, upper | 61982 | 20-09-25 |
| Imperial [13] | S6 ^(k)^ 3rd peak | Daily infections, mean | 354472 | 20-12-20 |
| Imperial [13] | S6 ^(k)^ 3rd peak | Daily infections, lower | 115631 | 21-01-03 |
| Imperial [13] | S6 ^(k)^ 3rd peak | Daily infections, upper | 717356 | 20-12-04 |
| LANL [14] | S1 ^(l)^ 1st peak | Daily infections, median | 2979 | 20-06-01 |
| LANL [14] | S1 ^(l)^ 1st peak | Daily infections, lower | 2979 | 20-06-01 |
| LANL [14] | S1 ^(l)^ 1st peak | Daily infections, upper | 2979 | 20-06-01 |
| LANL [14] | S1 ^(l)^ 2nd peak | Daily infections, median | 3825 | 20-10-01 |
| LANL [14] | S1 ^(l)^ 2nd peak | Daily infections, lower | 3825 | 20-10-01 |
| LANL [14] | S1 ^(l)^ 2nd peak | Daily infections, upper | 3825 | 20-10-01 |
| LANL [14] | S1 ^(l)^ 3rd peak | Daily infections, median | .. | .. |
| LANL [14] | S1 ^(l)^ 3rd peak | Daily infections, lower | .. | .. |
| LANL [14] | S1 ^(l)^ 3rd peak | Daily infections, upper | 6526 | 20-10-25 |
| Rahimi Rise [29] | S1 ^(p)^ Highest peak | Daily cases | 155297 | 20-03-31 |
| Rahimi Rise [29] | S2 ^(q)^ Highest peak | Daily cases | 470229 | 20-03-31 |
| Zareie [99] | S1 ^(l)^ 1st peak | Daily cases | 76020 | 20-10-06 |
|  |  | ***Incident Daily Cases, Confirmed and suspected*** |  |  |
| Saberi (paper) [22] | S2 ^(w)^ Single peak | Daily confirmed and suspected cases, mean | 2120450 | 20-05-13 |
| Saberi (paper) [22] | S2 ^(w)^ Single peak | Daily confirmed and suspected cases, lower | 1862460 | 20-05-13 |
| Saberi (paper) [22] | S2 ^(w)^ Single peak | Daily confirmed and suspected cases, upper | 2380785 | 20-05-13 |
| Saberi (paper) [22] | S3 ^(x)^ Single peak | Daily confirmed and suspected cases, mean | 422409 | 20-05-25 |
| Saberi (paper) [22] | S3 ^(x)^ Single peak | Daily confirmed and suspected cases, lower | 345012 | 20-05-25 |
| Saberi (paper) [22] | S3 ^(x)^ Single peak | Daily confirmed and suspected cases, upper | 502151 | 20-05-25 |
|  |  | ***Prevalent Daily Cases*** |  |  |
| Ghaffarzadegan [41] | S1P1 ^(y)^ 1st peak | Current cases | 492473 | 20-03-19 |
| Ghaffarzadegan [41] | S1P2 ^(z)^ 1st peak | Current cases | 492473 | 20-03-19 |
| Ghaffarzadegan [41] | S2P1 ^(aa)^ 1st peak | Current cases | 492473 | 20-03-19 |
| Ghaffarzadegan [41] | S2P2 ^(bb)^ 1st peak | Current cases | 492473 | 20-03-19 |
| Ghaffarzadegan [41] | S3P1 ^(cc)^ 1st peak | Current cases | 492473 | 20-03-19 |
| Ghaffarzadegan [41] | S3P2 ^(dd)^ 1st peak | Current cases | 492473 | 20-03-19 |
| Ghaffarzadegan [41] | S1P1 ^(y)^ 2nd peak | Current cases | 334801 | 20-05-08 |
| Ghaffarzadegan [41] | S2P1 ^(aa)^ 2nd peak | Current cases | 322697 | 20-05-03 |
| Ghaffarzadegan [41] | S3P1 ^(cc)^ 2nd peak | Current cases | 322697 | 20-05-03 |
| Gu (YYG) [17] | S1 ^(l)^ 1st peak | Daily prevalent cases, mean | 641888 | 20-03-14 |
| Gu (YYG) [17] | S1 ^(l)^ 1st peak | Daily prevalent cases, lower | 224066 | 20-03-13 |
| Gu (YYG) [17] | S1 ^(l)^ 1st peak | Daily prevalent cases, upper | 1229231 | 20-03-13 |
| Gu (YYG) [17] | S1 ^(l)^ 2nd peak | Daily prevalent cases, mean | 1729160 | 20-11-01 |
| Gu (YYG) [17] | S1 ^(l)^ 2nd peak | Daily prevalent cases, lower | 1064222 | 20-08-05 |
| Gu (YYG) [17] | S1 ^(l)^ 2nd peak | Daily prevalent cases, upper | 4620216 | 20-11-01 |
| Mashayekhi [28] | S1 ^(u)^ Single peak | Daily [symptomatic?] cases | 41429 | 20-03-18 |
| Mashayekhi [28] | S1 ^(m)^ Single peak | Daily asymptomatic cases | 160119 | 20-03-21 |
| Mashayekhi [28] | S2 ^(n)^ Single peak | Daily [symptomatic?] cases ^(y)^ | 258916 | 20-03-24 |
| Mashayekhi [28] | S2 ^(n)^ Single peak | Daily asymptomatic cases ^(z)^ | 294680 | 20-04-10 |
| Mashayekhi [28] | S3 ^(o)^ Single peak | Daily [symptomatic?] cases ^(aa)^ | 3510276 | 20-06-21 |
| Mashayekhi [28] | S3 ^(o)^ Single peak | Daily asymptomatic cases ^(bb)^ | 2407381 | 20-06-11 |
| Saberi (web site) [21] | S1P10 ^(ee)^ Single | Daily active cases | 2240000 | 20-06-19 |
| Saberi (web site) [21] | S1P30 ^(ff)^ Single | Daily active cases | 6709000 | 20-07-04 |
| Saberi (web site) [21] | S1P50 ^(gg)^ Single | Daily active cases | 11180000 | 20-07-10 |
| Saberi (web site) [21] | S1P80 ^(hh)^ Single | Daily active cases | 17880000 | 20-07-16 |
| Saberi (web site) [21] | S2P10 ^(ii)^ Single | Daily active cases | 2263000 | 20-05-29 |
| Saberi (web site) [21] | S2P30 ^(jj)^ Single | Daily active cases | 6731000 | 20-06-13 |
| Saberi (web site) [21] | S2P50 ^(kk)^ Single | Daily active cases | 11200000 | 20-06-19 |
| Saberi (web site) [21] | S2P80 ^(ll)^ Single | Daily active cases | 17900000 | 20-06-25 |
| Saberi (web site) [21] | S3P10 ^(mm)^ Single | Daily active cases | 2290000 | 20-05-19 |
| Saberi (web site) [21] | S3P30 ^(nn)^ Single | Daily active cases | 6759000 | 20-06-03 |
| Saberi (web site) [21] | S3P50 ^(oo)^ Single | Daily active cases | 11230000 | 20-06-10 |
| Saberi (web site) [21] | S3P80 ^(pp)^ Single | Daily active cases | 17930000 | 20-06-16 |
|  |  | ***Cumulative Cases Synchronous with Prevalent Daily Cases*** |  |  |
| Saberi (web site) [21] | S1P10 ^(ee)^ Single | Cumulative cases | 8837600 | 20-06-19 |
| Saberi (web site) [21] | S1P30 ^(ff)^ Single | Cumulative cases | 26503000 | 20-07-04 |
| Saberi (web site) [21] | S1P50 ^(gg)^ Single | Cumulative cases | 44168000 | 20-07-10 |
| Saberi (web site) [21] | S1P80 ^(hh)^ Single | Cumulative cases | 70665000 | 20-07-16 |
| Saberi (web site) [21] | S2P10 ^(ii)^ Single | Cumulative cases | 8857700 | 20-05-29 |
| Saberi (web site) [21] | S2P30 ^(jj)^ Single | Cumulative cases | 26523000 | 20-06-13 |
| Saberi (web site) [21] | S2P50 ^(kk)^ Single | Cumulative cases | 44188000 | 20-06-19 |
| Saberi (web site) [21] | S2P80 ^(ll)^ Single | Cumulative cases | 70685000 | 20-06-25 |
| Saberi (web site) [21] | S3P10 ^(mm)^ Single | Cumulative cases | 8882600 | 20-05-19 |
| Saberi (web site) [21] | S3P30 ^(nn)^ Single | Cumulative cases | 26548000 | 20-06-03 |
| Saberi (web site) [21] | S3P50 ^(oo)^ Single | Cumulative cases | 44213000 | 20-06-10 |
| Saberi (web site) [21] | S3P80 ^(pp)^ Single | Cumulative cases | 70711000 | 20-06-16 |
| Zhan [40] | S1 ^(l)^ Single | Cumulative cases, mean | 14450 | 20-03-22 |
| Zhan [40] | S1 ^(l)^ Single | Cumulative cases, lower | 8206 | 20-03-22 |
| Zhan [40] | S1 ^(l)^ Single | Cumulative cases, upper | 20694 | 20-03-22 |

* Peaks not mentioned in this table, i.e. peaks 1, 2, and 3 of IHME [12], and peaks 1 and 2 of Imperial [13], replicate or imitate official reports.

(a) N/A: Not applicable.

(b) As of 2020-10-19.

(c) S1: Best (Masks): ‘Universal Masks’ scenario reflects 95% mask usage in public in every location.

(d) S2: Reference (Current): ‘Current projection’ scenario assumes social distancing mandates are re-imposed for 6 weeks whenever daily deaths reach 8 per million (0.8 per 100,000).

(e) S3: Worse (Easing): ‘Mandates easing’ scenario reflects continued easing of social distancing mandates, and mandates are not re-imposed.

(f) S1: Additional 50% Reduction.

(g) S2: Maintain Status Quo.

(h) S3: Relax Interventions 50%.

(i) S4: Surged Additional 50% Reduction.

(j) S5: Surged Maintain Status Quo.

(k) S6: Surged Relax Interventions 50%.

(l) S1: Single scenario.

(m) S1: Serious distancing.

(n) S2: Not serious distancing.

(o) S3: Worse than Scenario 2.

(p) S1: Interventions.

(q) S2: No interventions.

(r) S1: Logistic regression

(s) S2: LSTM (Long Short-Term Memory)

(t) S1: Worst scenario, minimum (25%) isolation.

(u) S2: Medium scenario, medium (32%) isolation.

(v) S3: Best scenario, maximum (40%) isolation.

(w) S2: Current trend.

(x) S3: 20% less distancing.

(y) S1P1: Seasonality conditions 1 (no effect or status quo) and Policy effect 1 (status quo contact rate). Estimates for 2020-03-19, the end of first month after the epidemic start date, are equal across the six scenarios.

(z) S1P2: Seasonality conditions 1 (no effect or status quo) and Policy effect 2 (aggressive efforts to decrease contact rate by half of what it would be otherwise).

(aa) S2P1: Seasonality conditions 2 (moderate effect; infectivity of the virus decreases linearly from April 1st and halves by June 1st, then stays the same for the rest of the simulation) and Policy effect 1 (status quo contact rate).

(bb) S2P2: Seasonality conditions 2 (moderate effect; infectivity of the virus decreases linearly from April 1st and halves by June 1st, then stays the same for the rest of the simulation) and Policy effect 2 (aggressive efforts to decrease contact rate by half of what it would be otherwise).

(cc) S3P1: Seasonality conditions 3 (very strong mitigating effect; infectivity of the virus decreases from April 1st to a quarter of its base value by June 1st, then stays the same for the rest of the simulation) and Policy effect 1 (status quo contact rate).

(dd) S3P2: Seasonality conditions 3 (very strong mitigating effect; infectivity of the virus decreases from April 1st to a quarter of its base value by June 1st, then stays the same for the rest of the simulation) and Policy effect 2 (aggressive efforts to decrease contact rate by half of what it would be otherwise).

(ee) S1P10: Scenario 1 (Best scenario, based on official reports with correction factor of 1) with 10 million susceptible population.

(ff) S1P30:Scenario 1 (Best scenario, based on official reports with correction factor of 1) with 30 million susceptible population.

(gg) S1P50: Scenario 1 (Best scenario, based on official reports with correction factor of 1) with 50 million susceptible population.

(hh) S1P80: Scenario 1 (Best scenario, based on official reports with correction factor of 1) with 80 million susceptible population.

(ii) S2P10: Scenario 2 (Medium scenario, based on official reports with correction factor of 5 (after Dr. Rick Brennan, Director of Emergency Operations, World Health Organization [54]) with 10 million susceptible population.

(jj) S2P30: Scenario 2 (Medium scenario, based on official reports with correction factor of 5 (after Dr. Rick Brennan, Director of Emergency Operations, World Health Organization [54]) with 30 million susceptible population.

(kk) S2P50: Scenario 2 (Medium scenario, based on official reports with correction factor of 5 (after Dr. Rick Brennan, Director of Emergency Operations, World Health Organization [54]) with 50 million susceptible population.

(ll) S2P80: Scenario 2 (Medium scenario, based on official reports with correction factor of 5 (after Dr. Rick Brennan, Director of Emergency Operations, World Health Organization [54]) with 80 million susceptible population.

(mm) S3P10: Scenario 3 (Worst scenario, based on official reports with correction factor of 10 (after Russell [55]) with 80 million susceptible population.

(nn) S3P30: Scenario 3 (Worst scenario, based on official reports with correction factor of 10 (after Russell [55]) with 30 million susceptible population.

(oo) S3P50: Scenario 3 (Worst scenario, based on official reports with correction factor of 10 (after Russell [55]) with 50 million susceptible population.

(pp) S3P80: Scenario 3 (Worst scenario, based on official reports with correction factor of 10 (after Russell [55]) with 10 million susceptible population.

Appendix Table 5. Predictions of epidemic control dates and values of outcomes

| Study 1st author | Scenario / model | Outcome | Value | Date | Description |
| --- | --- | --- | --- | --- | --- |
| Ahmadi [44] | M1 ^(a)^ | Cumulative cases | 87000 | 20-05-13 | End of the epidemic |
| Ahmadi [44] | M2 ^(b)^ | Cumulative cases | 87000 | 20-05-13 | End of the epidemic |
| Ahmadi [44] | M1 ^(c)^ | Cumulative deaths | 11000 | 20-06-01 | End of the epidemic |
| Ahmadi [44] | M2 ^(d)^ | Cumulative deaths | 4900 | 20-06-01 | End of the epidemic |
| Haghdoost [27] | S2 ^(e)^ | Maximum number of infected people in day | 92100 | 20-04-20 to 20-05-20 | Epidemic is well controlled |
| Haghdoost [27] | S3 ^(6f^ | Maximum number of infected people in day | 9150 | 20-04-20 to 20-05-20 | Epidemic is well controlled |
| Zhan [40] | S0 ^(g)^ | ·· | ·· | By end of April 2020 | Epidemic under control |
| Zhan [40] | S0 ^(h)^ | ·· | ·· | Before June 2020 | Epidemic expected to end |

(a) Ahmadi, M1: Model 1, Gompertz [44].

(b) Ahmadi, M2: Model 2, Von Bertalanffy [44].

(c) Ahmadi, M1: Model 1, Gompertz [44].

(d) Ahmadi, M2: Model 2, Von Bertalanffy [44].

(e) Haghdoost, S2: Medium scenario, medium (32%) isolation [27].

(f) Haghdoost, S3: Best scenario, maximum (40%) isolation [27].

(g) Zhan, S0: Single scenario. Estimated outcome values not provided for “epidemic under control by the end of April 2020” [40].

(h) Zhan, S0: Single scenario. Estimated outcome values not provided for “epidemic expected to end before June 2020” [40].

Appendix Figure 1. PRISMA 2009 study flow diagram

## Screening

## Included

## Eligibility

## Identification

Records identified through database searching
(n = 139)

Additional records identified through other sources
(n = 15)

Records after duplicates removed
(n = 154)

Records screened
(n = 154)

Full-text articles assessed for eligibility
(n = 51)

Studies included in qualitative synthesis
(n = 29)

Records excluded (n = 85)

(no estimates for outcomes of interest)

Full-text articles excluded, with reasons (n = 22)

(3 Excluded due to absent minimum details,

19 Excluded for absence of estimates for outcomes of interest)

Appendix Figure 2. Officially reported cumulative confirmed cases, deaths, and recovered cases of COVID-19 in Iran

Appendix Figure 3. Reported daily confirmed cases, deaths, and recovered cases of COVID-19 in Iran

Appendix Figure 4. Reported and median-scenario estimated daily prevalent cases of COVID-19 in Iran, including predictions by Saberi

(1) Ghaffarzadegan S1P2: Seasonality conditions 1 (no effect or status quo) and Policy effect 2 (aggressive efforts to decrease contact rate by half of what it would be otherwise) [41]. (2) Haghdoost S2: Medium scenario, medium (32%) isolation [27]. (3) Mashayekhi S2: Medium scenario, not serious distancing; People reduce their social [physical] contacts only to 20% of regular level, voluntarily, after number of cases and deaths have increased, and other settings are like scenario one [28]. (4) Saberi S2P50: Scenario 2, Medium scenario, based on official reports with correction factor of 5 (after Dr. Rick Brennan, Director of Emergency Operations, World Health Organization [54]) with 50 million susceptible population [21]. (5) Cases CF 5: Reported cases with a Correction Factor of 5, after Dr. Rick Brennan, Director of Emergency Operations, World Health Organization [54]. (6) Cases CF 10: Reported cases with a Correction Factor of 10, after Russell [55]. (7) MOHME reported: Official reported cumulative cases minus cumulative deaths minus cumulative recovered via [4, 5].

Appendix Figure 5. Reported and median-scenario estimated daily prevalent case of COVID-19 in Iran, without predictions by Saberi

(1) Ghaffarzadegan S1P2: Seasonality conditions 1 (no effect or status quo) and Policy effect 2 (aggressive efforts to decrease contact rate by half of what it would be otherwise) [41]. (2) Haghdoost S2: Medium scenario, medium (32%) isolation [27]. (3) Mashayekhi S2: Medium scenario, not serious distancing; People reduce their social [physical] contacts only to 20% of regular level, voluntarily, after number of cases and deaths have increased, and other settings are like scenario one [28]. (4) Cases CF 5: Reported cases with a Correction Factor of 5, after Dr. Rick Brennan, Director of Emergency Operations, World Health Organization [54]. Cases (5) CF 10: Reported cases with a Correction Factor of 10, after Russell [55]. (6) MOHME reported: Official reported cumulative cases minus cumulative deaths minus cumulative recovered via [4, 5].

Appendix Figure 6. Reported and worst-scenario estimated cumulative deaths of COVID-19 in Iran, including predictions by Mashayekhi

(1) Ahmadi M3: Model 3, Cubic Polynomial [44]. (2) Ghaffarzadegan S1P1: Seasonality conditions 1 (no effect or status quo) and Policy effect 1 (status quo contact rate) [17]. (3) Haghdoost S1: Worst scenario, minimum (25%) isolation [27]. (4) Mashayekhi S3: Worst scenario. People reduce their social [physical] contacts only to 50% of regular level, voluntarily, after number of cases and deaths have increased, plus inadequate observation of sanitation cautions, so that transmission rate reduces only by 40% (instead of 55%), and 60% of people do not observe the sanitation cautions [28]. (5) Deaths CF 5: Reported deaths with a Correction Factor of 5, after Dr. Rick Brennan, Director of Emergency Operations, World Health Organization [54]. (6) Deaths CF 10: Reported deaths with a Correction Factor of 10, after Russell [55]. (7) MOHME reported: Official reported deaths via [4, 5].

Appendix Figure 7. Reported and worst-scenario estimated cumulative deaths of COVID-19 in Iran, without predictions by Mashayekhi

(1) Ahmadi M3: Model 3, Cubic Polynomial [44]. (2) Ghaffarzadegan S1P1: Seasonality conditions 1 (no effect or status quo) and Policy effect 1 (status quo contact rate) [41]. (3) Haghdoost S1: Worst scenario, minimum (25%) isolation [27]. (4) Deaths CF 5: Reported deaths with a Correction Factor of 5, after Dr. Rick Brennan, Director of Emergency Operations, World Health Organization [54]. (5) Deaths CF 10: Reported deaths with a Correction Factor of 10, after Russell [55]. (6) MOHME reported: Official reported deaths via [4, 5].

Appendix Figure 8. Reported and current (median) scenario estimated cumulative deaths of COVID-19 in Iran, International studies

~~~~

(1) DELPHI: DELPHI (Differential Equations Leads to Predictions of Hospitalizations and Infections) Epidemiological Case Predictions. Mean estimate [10]. (2) IHME: Institute for Health Metrics and Evaluation (IHME) Mean estimate [12]. (3) Imperial: Imperial College COVID-19 LMIC Reports. Mean estimate [13]. (4) LANL: Los Alamos National Laboratory (LANL) COVID-19 Cases and Deaths Forecasts. Mean estimate [14]. (5) Srivastava: ReCOVER- Accurate Predictions and Resource Management for COVID-19 Epidemic Response. Mean estimate [15]. (6) YYG (Youyang Gu): COVID-19 Projections Using Machine Learning. Mean estimate [17]. (7) MOHME reported: Ministry of Health and Medical Education, Iran [4, 5].
